# Laryngeal cancer incidence trends in the United States over 2000–2020: a population-based analysis

**DOI:** 10.1186/s13690-024-01333-1

**Published:** 2024-07-10

**Authors:** Seyed Ehsan Mousavi, Mehran Ilaghi, Armin Aslani, Morvarid Najafi, Zahra Yekta, Seyed Aria Nejadghaderi

**Affiliations:** 1 Neurosciences Research Center, Aging Research Institute, Tabriz University of Medical Sciences, Tabriz, Iran; 2 Department of Community Medicine, Social Determinants of Health Research Center, Faculty of Medicine, Tabriz University of Medical Sciences, Tabriz, Iran; 3 Institute of Neuropharmacology, Kerman Neuroscience Research Center, Kerman University of Medical Sciences, Kerman, Iran; 4 Center for Orthopedic Trans-Disciplinary Applied Research, Tehran University of Medical Sciences, Tehran, Iran; 5 Calaveras County Department of Health, Calaveras County, San Andreas, CA USA; 6 HIV/STI Surveillance Research Center, WHO Collaborating Center for HIV Surveillance, Institute for Futures Studies in Health, Kerman University of Medical Sciences, Kerman, Iran; 7 Systematic Review and Meta‑analysis Expert Group (SRMEG), Universal Scientific Education and Research Network (USERN), Tehran, Iran

**Keywords:** Laryngeal Cancer, Head and Neck Cancer, Epidemiology, United States, Surveillance, epidemiology, and end results, SEER, Incidence

## Abstract

**Introduction:**

Laryngeal cancers account for one-third of all head and neck cancers. We aimed to report the incidence trends of laryngeal cancer over 2000–2020 in the United States (US), by age, sex, race/ethnicity, and histological subtypes.

**Methods:**

Data from the Surveillance, Epidemiology, and End Results 22 database were used to identify patients with laryngeal cancer based on the International Classification of Diseases for Oncology, version 3. Age-standardized incidence rates (ASIRs) for laryngeal cancer, adjusted for reporting delays, were calculated. The Joinpoint Regression Program was then utilized to determine annual percent changes (APCs) and average annual percent changes (AAPCs) in the trends. The analysis excluded data from 2020 to prevent potential bias related to the COVID-19 pandemic.

**Results:**

A total of 104,991 cases of laryngeal cancer were identified in the US from 2000 to 2019. Squamous cell carcinoma was the predominant subtype, accounting for 94.53% of cases. Above 73.20% occurred among non-Hispanic whites, with the highest incidence observed among individuals aged 55–69 years (46.71%). The ASIRs were 5.98 and 1.25 per 100,000 population for men and women, respectively. Over 2000–2019, there was a significant reduction in ASIRs for laryngeal cancer in both sexes. Non-Hispanic black men exhibited the highest ASIR (9.13 per 100,000) and the largest decline in the ASIRs over 2000–2019 (AAPC: -3.26%).

**Conclusions:**

Laryngeal cancer incidence rates showed a decline from 2000 to 2019, in addition to 2020, during the COVID-19 pandemic. Additional research is required to investigate risk factors and their influence on incidence rates of laryngeal cancer.

**Supplementary Information:**

The online version contains supplementary material available at 10.1186/s13690-024-01333-1.

**Table:** 

**Text box 1. Contributions to the literature**	
• This provides a comprehensive analysis of laryngeal cancer incidence trends in the US from 2000 to 2019, highlighting variations by age, sex, race/ethnicity, and histological subtypes.	
• The study demonstrates a significant decline in age-standardized incidence rates (ASIRs) for laryngeal cancer across all demographic groups, with the most pronounced reduction observed in non-Hispanic black men.	
• The study enhances the understanding of demographic disparities in laryngeal cancer incidence, emphasizing the need for targeted public health interventions and further research on underlying risk factors.	

## Introduction

Globally, head and neck cancers have been led to higher than 660 and 325 thousands of incident and mortality cases, respectively [1]. These malignancies can originate in diverse anatomical regions, including the oral cavity, pharynx, larynx, nasal cavity, paranasal sinuses, thyroid, and salivary glands [2, 3]. Despite an overall decrease in the burden of laryngeal cancer in recent decades, it is estimated that global incidence of head and neck cancer will increase by 30% by 2030 [1, 4]. Laryngeal cancers comprise one-third of all head and neck cancers, with squamous cell carcinomas (SCCs) accounting for most of these malignancies [1, 5]. Tobacco and alcohol consumption are the major risk factor for laryngeal cancer [4].

In the United States (US), laryngeal cancer accounted for 12,380 new cases and 3,820 deaths in 2023 [6]. This cancer displays significant disparities based on race and sex, with a higher incidence in men than women (9,000 vs. 2,480) and a higher survival rates in Whites than Blacks (62% vs. 53%) [6]. Existing literature in the US has reported a steadily decreasing incidence and mortality rates for laryngeal cancer [7, 8]. Despite this overall decrease in incidence, the five-year survival rate of laryngeal cancer has declined over the past four decades, dropping from 66 to 61% [6].

COVID-19 has significantly impacted non-COVID care, patient referrals, and identification. In this regard, there was a decrease in incidences of oral cavity and laryngeal carcinomas during April and May 2020, in addition to a 1.9% reduction in the number of patients starting cancer treatment in 2020 compared to the pre-pandemic period [9, 10].

Prior investigations on laryngeal cancer incidence trends have primarily focused on specific histological subtypes, thus offering a limited perspective. Moreover, these studies predominantly cover earlier timeframes, and none have assessed the influence of the COVID-19 pandemic on laryngeal cancer trends [11–14]. Hence, the objective of this study was to evaluate the effects of COVID-19 and report trends of laryngeal cancer incidence rates among diverse demographic and morphological subcategories within the US over 2000–2020, using the Surveillance, Epidemiology, and End Results (SEER) data.

## Methods

### Data source

The SEER Program is an extensive population-based archive of cancer-related information within the US. SEER 22, which encapsulates nearly 48% of the American populace, furnishes statistics about patient survival rates and cancer staging at the point of initial diagnosis. Patients’ demographic attributes, the initial anatomical site of the tumor, the histological characteristics of the cancer, the stage at which the diagnosis occurred, the initial therapeutic interventions, and the ongoing surveillance of vital status are all encompassed within the data compiled by the SEER program [15]. In this investigation, the SEER 22 database, accessible since April 2023 and comprising data reported up to November 2022, was utilized to calculate the incidence rates and annual percent changes (APCs) of laryngeal cancer over 2000–2020 [16, 17]. Access to the SEER 22 database was conducted in adherence to the SEER Research Data Agreement for 1975–2020 Data [18], and the presentation of cancer statistics followed the SEER 22 guidelines [19].

### Definitions

Cancer incidence is shown using frequencies and percentages, with rates indicated as cases per 100,000 individuals. APCs in laryngeal cancer over a specific time frame exhibit fluctuations in proportion to the rate observed in the previous year. The average annual percent changes (AAPCs) represent the mean of multiple APCs during a designated period. Participants were categorized into three groups by race/ethnicity: Non-Hispanic White (NHW), Non-Hispanic Black (NHB), and Hispanic. Limited case numbers resulted in the race and ethnicity groups of American Indian/Alaska Native, Native Hawaiian, and Asian/Pacific Islander solely being utilized for calculating parameters related to all races and ethnicities. Laryngeal cancer patients were identified using the International Classification of Diseases for Oncology version 3. Laryngeal cancer morphologies were classified as chondrosarcoma (code 9220), neuroendocrine carcinoma (codes 8013, 8041, 8240, 8246, and 8249), and SCC (codes 8051, 8052, 8070–8072, 8074, 8076, 8082, 8083, and 8560).

### Statistical analysis

This study used the SEER 22 Research Limited-Field Data with Delay-Adjustment database from 2000 to 2020 to calculate the delay-adjusted age-standardized incidence rate (ASIR) of laryngeal cancer. The data were obtained using SEER*Stat software, version 8.4.1.2 [20]. The objective behind modeling reporting delay is to accommodate anticipated future data revisions [21]. These modified counts and the delay model can improve the accuracy [21]. The inclusion criteria were restricted to individuals with a confirmed cancer diagnosis where the age at diagnosis was known. Subsequently, a delay model was applied, incorporating adjustment factors such as cancer site, registry, age group, race and ethnicity, and year of diagnosis [22, 23].

Additionally, to determine the ASIR of laryngeal cancer subtypes, the study utilized the SEER 22 Research Limited-Field Data database for the years 2000 to 2020 [17], which was accessed through SEER*Stat, version 8.4.1.2 [20]. The Tiwari technique was employed for estimating the ASIRs regarding the 2000 US standard population and calculating the associated 95% confidence intervals (CIs) using SEER*Stat version 8.4.1.2 [20, 24]. The Joinpoint Regression Program, version 5.0.2 [25], was employed to estimate the APCs and AAPCs [26] for ASIRs [27] through the utilization of joinpoint regression modeling and parallelism test [28]. The year 2020 marked the inception of the COVID-19 pandemic. Given the potential bias introduced by the 2020 incidence data into cancer incidence estimates, these data were deliberately omitted from the Joinpoint trend analysis and were solely presented in graphical form. The computation of the APCs for the ASIR of laryngeal cancer involved fitting the best-fitting least-squares regression lines to the natural logarithm of the ASIR; in this regression, the year of diagnosis served as the regressor variable. Specific criteria were established: a minimum of two observations between two joinpoints and a minimum of two observations from the joinpoint to either end of the dataset. Model selection was accomplished using the weighted Bayesian Information Criteria approach [29]. The empirical quantile method was employed to determine the 95% CIs of AAPCs [30]. Additionally, to evaluate whether the trends in the two groups were similar over time, a pairwise comparison using the parallelism test was performed [28].

## Results

### Laryngeal cancer

#### Overall incidence

**Fig. 1:**
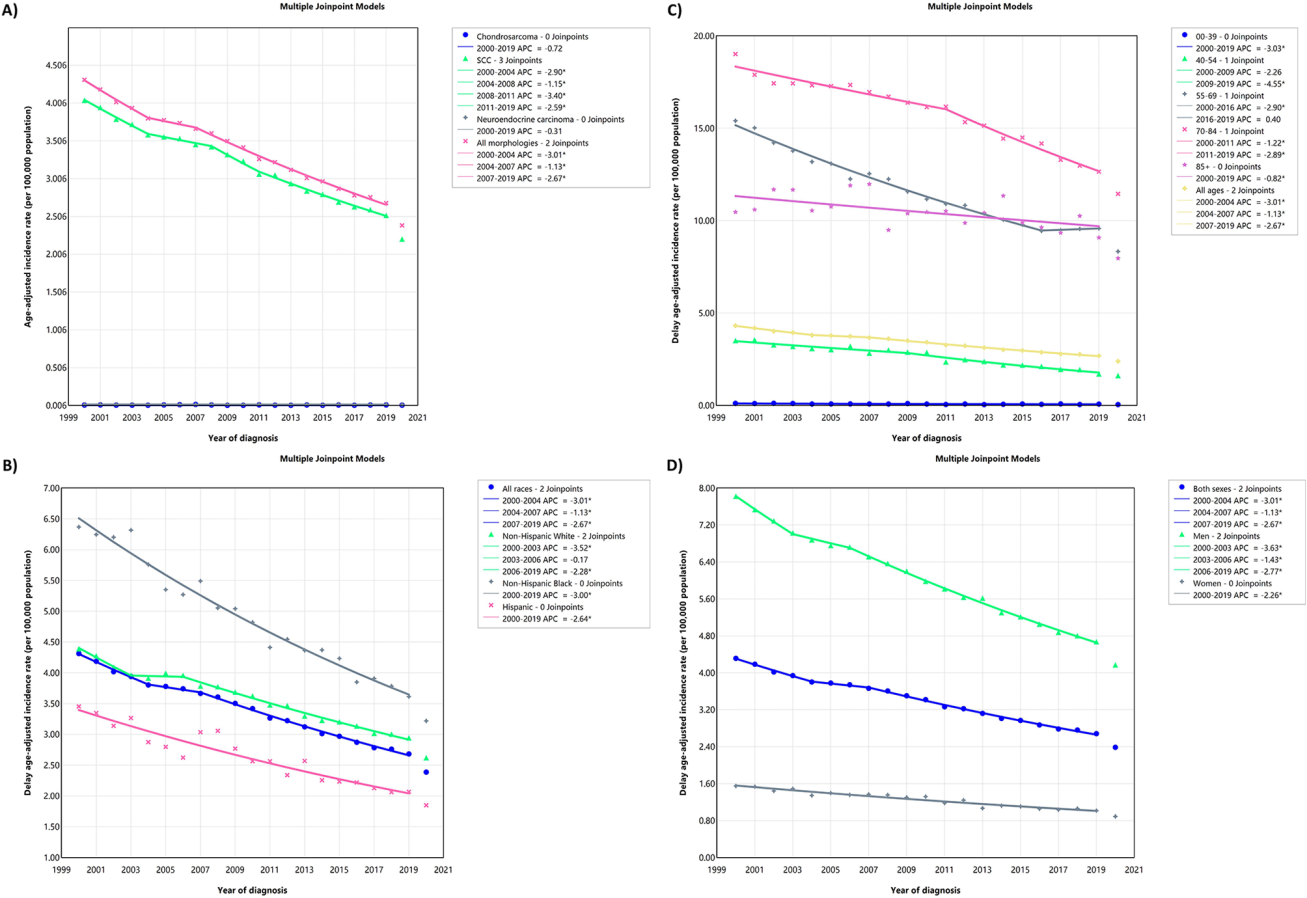
Delayed age-adjusted incidence rate of laryngeal cancer over 2000–2019 and in 2020 in the United States, by cancer subtypes **(A)**, race **(B)**, age **(C)**, and sex **(D)**. APC: annual percent change. * Represent p-value less than 0.05

From 2000 to 2019, a total of 104,991 cases of laryngeal cancer were documented in the US across all age groups. The most frequent subtype was SCC accounting for 94.53% of cases. Most cases were observed among NHWs (73.20%) and individuals aged 55–69 years (46.71%). The ASIRs were 5.98 (95% CI: 5.94, 6.02) and 1.25 (95% CI: 1.23, 1.27) per 100,000 for men and women, respectively. Notably, NHB men exhibited the highest ASIR at 9.13 (8.96, 9.31). Over 2000–2019, there was a significant reduction in ASIRs for laryngeal cancer (AAPC: -2.50% [-2.58, -2.43]). By sex, the AAPC was − 2.70% (-2.79, -2.61) for males and it was − 2.26% (-2.60, -1.93) for females. NHB men displayed the most significant decrease in ASIRs over the period 2000–2019 (AAPC: -3.26% [-3.60, -2.92]) (Table 1). Figure 1A, B and C, and 1D represent the delay-adjusted ASIR of laryngeal cancer by histological subtypes, race, age, and sex, respectively.

**Table 1:** Counts and age-standardized rate of laryngeal cancer incidence per 100,000 and average annual percent change from 2000 to 2019 in the United States, by age, sex, and race

**All race/ethnicities**						
**Age group (years)**	**Men**			**Women**		
	**Case (%)**	**Delayed ASIR (95% CI)**	**AAPC (95% CI)**	**Case (%)**	**Delayed ASIR (95% CI)**	**AAPC (95% CI)**
**All**	84,088 (80.09)	5.98 (5.94, 6.02)	-2.7 (-2.79, -2.61)	20,903 (19.91)	1.25 (1.23, 1.27)	-2.26 (-2.6, -1.93)
**0 to 39**	754 (0.72)	0.1 (0.09, 0.11)	-2.88 (-5.19, -0.79)	448 (043)	0.06 (0.05, 0.06)	-1.85 (-4.88, 0.29)
**40 to 54**	13,283 (12.65)	4.2 (4.13, 4.28)	-3.81 (-4.8, -3.11)	3871 (3.96)	1.2 (1.16, 1.24)	-2.45 (-4.53, -0.76)
**55 to 69**	39,538 (37.66)	19.25 (19.06, 19.45)	-2.55 (-2.81, -2.39)	9506 (9.05)	4.21 (4.12, 4.29)	-1.99 (-2.44, -1.59)
**70 to 84**	26,458 (25.20)	29.71 (29.36, 30.07)	-2.23 (-2.46, -2.02)	6056 (5.77)	5.17 (5.04, 5.3)	-2.24 (-2.91, -1.58)
**+ 85**	4055 (3.86)	25.45 (24.67, 26.24)	-1.85 (-2.36, -1.32)	1022 (0.97)	3.13 (2.94, 3.32)	-1.3 (-3.28, 0.74)
**Hispanic**						
**Age groups**	**Men**			**Women**		
	**Case (%)**	**Delayed ASIR (95% CI)**	**AAPC (95% CI)**	**Case (%)**	**Delayed ASIR (95% CI)**	**AAPC (95% CI)**
**All**	8586 (85.20)	5 (4.88, 5.11)	-2.9 (-3.45, -2.31)	1491 (14.80)	0.66 (0.63, 0.7)	-3.24 (-3.93, -2.06)
**0 to 39**	145 (1.44)	0.07 (0.06, 0.08)	-1.42 (-4.64, 1.99)	99 (0.98)	0.05 (0.04, 0.06)	-2.65 (-5.4, 0.16)
**40 to 54**	1538 (15.26)	2.62 (2.49, 2.76)	-4.82 (-6.3, -3.36)	329 (3.26)	0.56 (0.5, 0.62)	-3.52 (-5.71, -1.21)
**55 to 69**	3849 (38.20)	14.25 (13.8, 14.72)	-3.19 (-3.98, -2.32)	608 (6.03)	1.97 (1.81, 2.13)	-4.1 (-5.73, -1.09)
**70 to 84**	2673 (26.53)	29.06 (27.96, 30.19)	-2.31 (-3.38, -1.12)	375 (3.72)	2.95 (2.66, 3.26)	-2.49 (-3.87, -0.97)
**+ 85**	381 (3.78)	27.75 (25.03, 30.68)	-2.36 (-4.09, -0.2)	80 (0.79)	3.14 (2.49, 3.91)	N/A
**NHB**						
**Age groups**	**Men**			**Women**		
	**Case (%)**	**Delayed ASIR (95% CI)**	**AAPC (95% CI)**	**Case (%)**	**Delayed ASIR (95% CI)**	**AAPC (95% CI)**
**All**	11,756 (79.29)	9.13 (8.96, 9.31)	-3.26 (-3.6, -2.92)	3070 (20.71)	1.74 (1.68, 1.8)	-2.49 (-3.46, -1.52)
**0 to 39**	93 (0.63)	0.11 (0.09, 0.13)	N/A	47 (0.32)	0.05 (0.04, 0.07)	N/A
**40 to 54**	2235 (15.07)	6.49 (6.22, 6.77)	-5.39 (-6.62, -4.35)	706 (4.76)	1.81 (1.68, 1.95)	-4.58 (-7.13, -2.34)
**55 to 69**	6208 (41.87)	31.94 (31.15, 32.75)	-3.51 (-4.09, -2.91)	1557 (10.50)	6.38 (6.07, 6.71)	-2.06 (-3.11, -0.91)
**70 to 84**	2922 (19.71)	43.07 (41.51, 44.68)	-2.85 (-3.44, -2.32)	676 (4.56)	6.36 (5.89, 6.86)	-1.76 (-3.2, -0.29)
**+ 85**	298 (2.01)	31.34 (27.88, 35.11)	-1.61 (-4.26, 1.51)	84 (0.57)	3.49 (2.78, 4.32)	0.74 (-2.45, 4.78)
**NHW**						
**Age groups**	**Men**			**Women**		
	**Case (%)**	**Delayed ASIR (95% CI)**	**AAPC (95% CI)**	**Case (%)**	**Delayed ASIR (95% CI)**	**AAPC (95% CI)**
**All**	60,977 (79.34)	6.16 (6.11, 6.21)	-2.42 (-2.52, -2.29)	15,881 (20.66)	1.4 (1.38, 1.43)	-1.76 (-2.14, -1.39)
**0 to 39**	484 (0.63)	0.12 (0.11, 0.13)	-2.69 (-5.47, -0.35)	287 (0.37)	0.07 (0.06, 0.08)	-0.93 (-3.7, 1.24)
**40 to 54**	9075 (11.81)	4.64 (4.54, 4.73)	-2.79 (-4.1, -1.9)	2765 (3.60)	1.42 (1.36, 1.47)	-1.01 (-2.57, 0.3)
**55 to 69**	28,254 (36.76)	19.77 (19.54, 20.01)	-2.08 (-2.38, -1.9)	7152 (9.31)	4.7 (4.59, 4.81)	-1.7 (-2.25, -1.21)
**70 to 84**	19,942 (25.95)	29.87 (29.46, 30.29)	-2.08 (-2.56, -1.64)	4857 (6.32)	5.69 (5.53, 5.85)	-2.03 (-2.77, -1.36)
**+ 85**	3222 (4.19)	25.68 (24.8, 26.58)	-1.69 (-2.44, -0.91)	820 (1.70)	3.16 (2.95, 3.38)	-0.41 (-2.42, 1.51)

From 2015 to 2019, there were 25,725 cases of laryngeal cancer in the US. The majority of cases were observed among NHWs (70.86%) and those aged 55–69 years (49.68%). The ASIRs between 2015 and 2019 were 4.92 (4.85, 4.99) per 100,000 for men and 1.05 (1.03, 1.08) per 100,000 for women. Among all racial/ethnic groups, NHB men had the highest ASIR (7.22 [6.94, 7.51]). There was a decrease in ASIRs over the 2015–2019 period in both men and women with an AAPC of -2.77% (-2.94, -2.67) and − 2.26% (-2.60, -1.93), respectively. Additionally, NHB men exhibited the most substantial decline in ASIRs between 2015 and 2019 (AAPC: -3.26% [-3.60, -2.92]) (Table S1).

Table S2 displays the parallel trends of laryngeal cancer during the period from 2000 to 2019.

#### Men

Over 2000–2019, total number of 84,088 cases (80.09%) were diagnosed in men. SCC (94.83%) represented the most frequent subtype. Most cases were in the age group of 55–69 years (47.02%), primarily among NHWs (72.52%). Notably, cases aged 70–84 years exhibited the highest ASIR among all age groups, recording a rate of 29.71 (29.36, 30.07). The incidence rates had a consistent decrease across all age groups during the 2000–2019 period, with the age group of 40–54 years had the largest decline (AAPC: -3.81% [-4.80, -3.11]) (Table 1).

Among all reported cases, 10.21% were among Hispanic men, and SCC (93.79%) was the most common subtype. The majority of cases occurred between the ages of 55 and 69 years (44.83%). The ASIR in Hispanic cases was 5.00 (4.88, 5.11) with those between 70 and 84 years having the highest delayed ASIR (29.06 [27.96, 30.19]). Hispanic individuals exhibited a significant decrease in incidence rate with an AAPC of -2.90% (-3.45, -2.31) with those aged 40–54 years having the most substantial decrease in ASIR from 2000 to 2019 (AAPC: -4.82% [-6.30, -3.36]) (Table 1).

Of all males with laryngeal cancer, 13.98% were among NHB. SCC constituted 95.13% of reported cases among NHB men, with the majority (52.80%) falling between 55 and 69 years. The delayed ASIR per 100,000 population was 9.13 (8.96, 9.31), with cases aged 70–84 years had the highest delayed ASIR (43.07 [41.51, 44.68]). There was a significant decrease in ASIR among NHB men over 2000–2019 (AAPC: -3.26% [-3.60, -2.92]), with those aged 40–54 years had the highest decline (AAPC: -5.39% [-6.62, -4.35]) (Table 1).

In NHW men, SCC was the most prevalent subtype (94.92%). The majority of NHW men were 55–69 years old (46.34%), with the highest delayed ASIR in the 70–84 age group (29.87 [29.46, 30.29]). The overall AAPC showed a decline (-2.42% [-2.52, -2.29]) with cases between 40 and 54 years having the most largest drop in ASIR compared to other age groups (AAPC: -2.79% [-4.10, -1.90]) (Table 1).

#### Women

From 2000 to 2019, a total of 20,903 (19.91%) cases of laryngeal cancer were documented among women in the US. Predominant number of cases were SCC (93.32%). The majority occurred among NHWs (75.97%) and individuals aged 55–69 years (45.48%). Those between 70 and 84 years had the highest ASIR compared to the other age groups (5.17 [5.04, 5.30]) and those in 40–54 age group had the largest decrease in ASIRs over 2000–2019 (AAPC: -2.45% [-4.53, -0.76]) (Table 1).

Of all female cases, 7.13% were among Hispanic women, with SCC (91.15%) and aged 55–69 years (40.78%) as the most common subtype and age group, respectively. The delayed ASIR for Hispanic women was (0.66 [0.63, 0.70]) per 100,000. Those > 85 years had the highest ASIR (3.14 [2.49, 3.91]). Over 2000–2019, there was a decrease in the ASIRs of laryngeal cancer in Hispanic women (-3.24% [-3.93. -2.06]). Individuals aged 55–69 years showed the largest decline in incidence rate over 2000–2019 (-4.10% [-5.73. -1.09]) (Table 1).

Among females, 14.69% of cases were NHB, with SCC as the most common subtype (94.13%). The majority of them was in those aged 55–69 years (50.72%). The delayed ASIR in NHB women was 1.74 (1.68, 1.80) per 100,000. Cases aged 55–69 years exhibited the highest reported ASIR (6.38 [6.07, 6.71]). NHBs experienced a significant fall in incidence rate over 2000 to 2019 (AAPC: -2.49% [-3.46, -1.52]) with individuals aged 40–54 years had the largest decrease (-4.58% [-7.13. -2.34]) (Table 1).

The majority numbers of female cases were among NHWs. SCC was the most common subtype (93.41%) and the highest numbers were in those aged 55–69 years (45.03%). The delayed ASIR in NHW women was 1.40 (1.38, 1.43) per 100,000. Those aged 70–84 years demonstrated the highest reported ASIR (5.69 [5.53, 5.85]). NHW women had a substantial decrease in ASIRs with an AAPC of -1.76% (-2.14. -1.39) and those in 70–84 age group experienced the largest decline in ASIR compared to other age groups (AAPC: -2.03% [-2.77. -1.36]) (Table 1).

#### Age and sex patterns

**Fig. 2:**
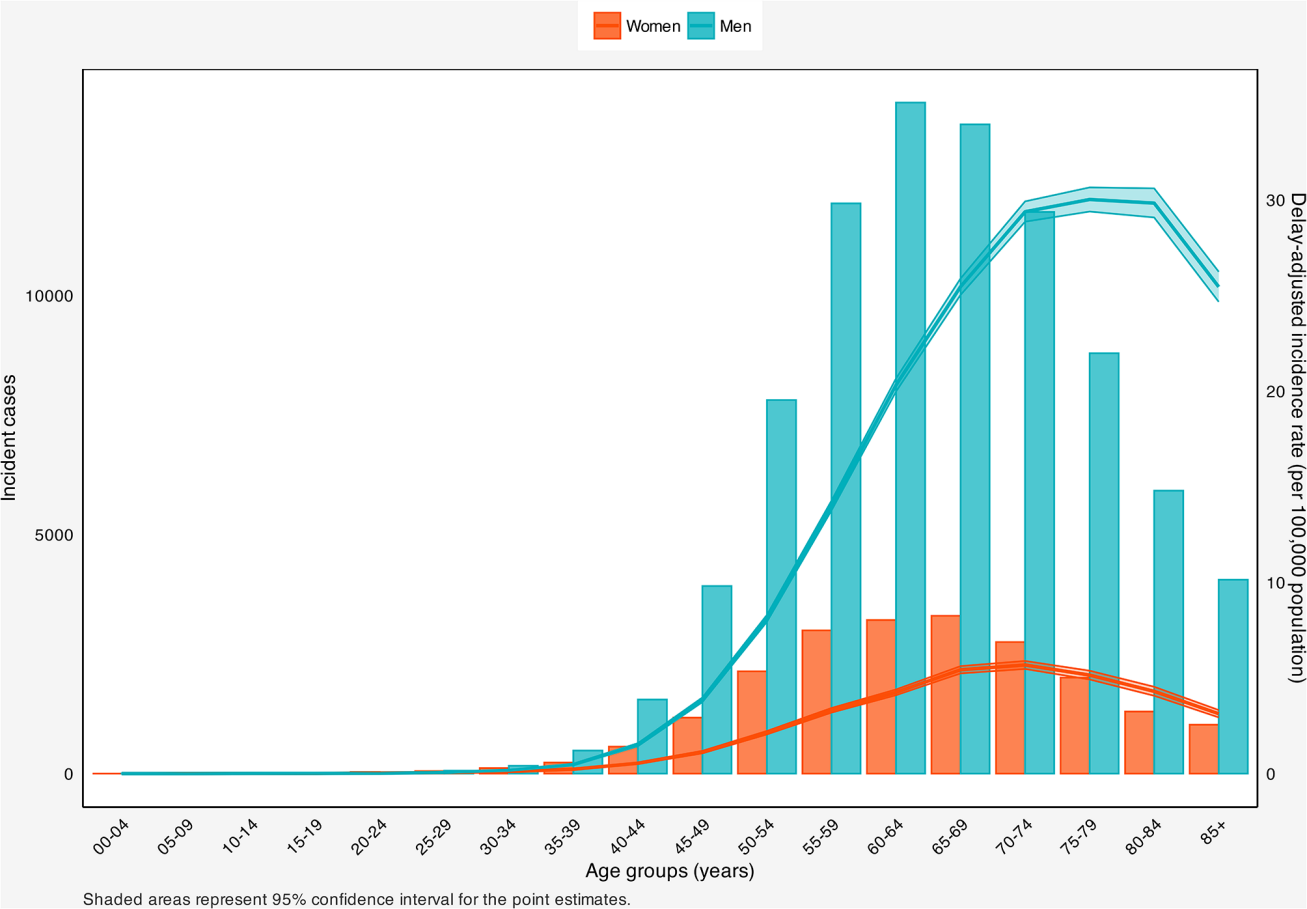
Number of incident cases and delay-adjusted incidence rate of laryngeal cancer in the United States among males and females in each age group. Shaded areas are the confidence interval range for the point estimates

Across all racial/ethnic groups, laryngeal cancer cases were scarcely reported up to 30–34 years. Subsequently, there was a marked surge in laryngeal cancer incident cases across both sexes, with men peaking in 60–64 and women in 65–69 years. Then, both sexes experienced a decline. Regarding the incidence rate, minimal fluctuations were observed until the 30–34 age group, after which both sexes experienced a substantial increase in incidence rates, reaching its peak between the ages of 75–79 for men and 70–74 for women. In all age groups, men consistently had higher incident numbers and rates (Fig. 2).

#### Coronavirus disease 2019 impacts

There was a notable decline in the ASIRs all races and ethnicities, for both males and females, and across all age groups (-11.02% [-14.64, -7.40]) for both sexes, and in women (-12.32% [-20.34, -4.30]) and men (-10.76% [-14.84, -6.69]) from 2019 to November 2020 (Table 2).

**Table 2:** Percent change in delay-adjusted age-standardized incidence rates of laryngeal cancer from 2019 to 2020 in all ages, by race and sex, using the November 2022 data

Races/ ethnicities	Sex	2019 Delayed ASIR (95% CI)	2020 Delayed ASIR (95% CI)	PC (95% CI)
All	Both	2.68 (2.61, 2.76)	2.39 (2.32, 2.46)	-11.02 (-14.64, -7.4)
	Female	1.02 (0.95, 1.08)	0.89 (0.83, 0.95)	-12.32 (-20.34, -4.3)
	Male	4.67 (4.53, 4.82)	4.17 (4.03, 4.31)	-10.76 (-14.84, -6.69)
Hispanic	Both	2.07 (1.9, 2.25)	1.85 (1.69, 2.02)	-10.61 (-21.41, 0.18)
	Female	0.59 (0.48, 0.72)	0.46 (0.36, 0.57)	-22.86 (-46.82, 1.1)
	Male	3.93 (3.57, 4.32)	3.56 (3.23, 3.91)	-9.51 (-21.66, 2.64)
NHB	Both	3.61 (3.35, 3.9)	3.22 (2.97, 3.49)	-10.91 (-20.69, -1.12)
	Female	1.34 (1.13, 1.58)	1.16 (0.97, 1.38)	-13.51 (-34.11, 7.09)
	Male	6.75 (6.18, 7.37)	6.05 (5.51, 6.63)	-10.39 (-21.73, 0.95)
NHW	Both	2.94 (2.84, 3.04)	2.62 (2.53, 2.72)	-10.97 (-15.37, -6.58)
	Female	1.21 (1.12, 1.31)	1.09 (1.01, 1.18)	-9.87 (-19.71, -0.02)
	Male	4.94 (4.76, 5.13)	4.39 (4.21, 4.57)	-11.22 (-16.14, -6.3)

**Abbreviations**: NHW: Non-Hispanic White; NHB: Non-Hispanic Black; ASIR: Age-standardized incidence rate; CI: Confidence interval, PC: percent change

### Squamous cell carcinoma

#### Overall incidence

Between 2000 and 2019, there were 99,247 cases of SCC in all age groups in the US. The majority of cases were in men (80.34%), NHWs (73.27%), and aged 55–69 years (47.20%). The ASIRs were 5.65 (5.61, 5.69) for men and 1.16 (1.15, 1.18) for women. The AAPCs for men and women were − 2.69% (-2.79, -2.56) and − 2.23% (-2.57, -1.90), respectively. NHB men had the highest ASIR (8.62; [8.45, 8.79]) and Hispanic women had the largest decrease in ASIRs (AAPC: -3.62% [-4.41, -2.23]) (Table 3, Figure S1, Figure S2, and Figure S3).

**Table 3:** Counts and age-standardized rate of laryngeal squamous cell carcinoma incidence per 100,000 and average annual percent change from 2000 to 2019 in the United States, by age, sex, and race

**All race/ethnicities**						
**Age group (years)**	**Men**			**Women**		
	**Case (%)**	**ASIR (95% CI)**	**AAPC (95% CI)**	**Case (%)**	**ASIR (95% CI)**	**AAPC (95% CI)**
**All**	79,740 (80.34)	5.65 (5.61, 5.69)	-2.69 (-2.79, -2.56)	19,507 (19.66)	1.16 (1.15, 1.18)	-2.23 (-2.57, -1.9)
**0 to 39**	668 (0.67)	0.09 (0.08, 0.09)	-2.72 (-5.41, -0.29)	385 (0.39)	0.05 (0.04, 0.05)	-2.73 (-5.89, -0.48)
**40 to 54**	12,739 (12.84)	4.03 (3.96, 4.1)	-3.8 (-4.77, -3.1)	3662 (3.69)	1.13 (1.09, 1.17)	-2.37 (-4.3, -0.78)
**55 to 69**	37,815 (38.10)	18.39 (18.2, 18.57)	-2.57 (-2.83, -2.4)	9027 (9.10)	3.99 (3.91, 4.07)	-2.01 (-2.53, -1.61)
**70 to 84**	24,926 (25.12)	27.94 (27.59, 28.29)	-2.14 (-2.58, -1.74)	5586 (5.63)	4.76 (4.64, 4.89)	-2.19 (-2.8, -1.6)
**+ 85**	3592 (3.62)	22.51 (21.78, 23.25)	-2.07 (-2.74, -1.35)	847 (0.85)	2.59 (2.41, 2.77)	0.13 (-2.17, 2.58)
**Hispanic**						
**Age groups**	**Men**			**Women**		
	**Case (%)**	**ASIR (95% CI)**	**AAPC (95% CI)**	**Case (%)**	**ASIR (95% CI)**	**AAPC (95% CI)**
**All**	8053 (85.56)	4.66 (4.55, 4.77)	-2.96 (-3.5, -2.39)	1359 (14.44)	0.6 (0.57, 0.64)	-3.62 (-4.41, -2.23)
**0 to 39**	125(1.33)	0.06 (0.05, 0.07)	-1.87 (-6.29, 2.8)	81(0.86)	0.04 (0.03, 0.05)	-3.76 (-7.33, -0.33)
**40 to 54**	1463(15.54)	2.49 (2.36, 2.62)	-4.91 (-6.46, -3.41)	301(3.20)	0.51 (0.45, 0.57)	-3.85 (-6.19, -1.46)
**55 to 69**	3628(38.55)	13.41 (12.97, 13.86)	-3.28 (-3.99, -2.49)	562(5.97)	1.81 (1.67, 1.97)	-4.29 (-5.94, -0.8)
**70 to 84**	2506(26.63)	27.18 (26.12, 28.28)	-2.34 (-3.31, -1.26)	349(3.71)	2.74 (2.46, 3.04)	-2.5 (-4.05, -0.81)
**+ 85**	331(3.52)	24.04 (21.52, 26.78)	-2.32 (-3.89, -0.37)	66(0.70)	2.58 (2, 3.28)	N/A
**NHB**						
**Age groups**	**Men**			**Women**		
	**Case (%)**	**ASIR (95% CI)**	**AAPC (95% CI)**	**Case (%)**	**ASIR (95% CI)**	**AAPC (95% CI)**
**All**	11,184(79.47)	8.62 (8.45, 8.79)	-3.31 (-3.65, -2.97)	2890(20.53)	1.63 (1.57, 1.69)	-2.47 (-3.46, -1.49)
**0 to 39**	79(0.56)	0.09 (0.07, 0.11)	N/A	37(0.26)	0.04 (0.03, 0.05)	N/A
**40 to 54**	2127(15.11)	6.16 (5.9, 6.43)	-5.39 (-6.61, -4.35)	669(4.75)	1.71 (1.58, 1.85)	-4.5 (-7.25, -2.07)
**55 to 69**	5963(42.37)	30.59 (29.81, 31.38)	-3.54 (-4.2, -2.86)	1486(10.56)	6.08 (5.77, 6.39)	-1.91 (-2.97, -0.74)
**70 to 84**	2753(19.56)	40.33 (38.82, 41.88)	-2.82 (-3.89, -1.79)	625(4.44)	5.85 (5.4, 6.33)	-1.84 (-3.79, 0.19)
**+ 85**	262(1.86)	27.44 (24.22, 30.98)	-2.83 (-5.67, 0.29)	73(0.52)	3.02 (2.37, 3.8)	1.24 (-1.88, 5.34)
**NHW**						
**Age groups**	**Men**			**Women**		
	**Case (%)**	**ASIR (95% CI)**	**AAPC (95% CI)**	**Case (%)**	**ASIR (95% CI)**	**AAPC (95% CI)**
**All**	57,882(79.60)	5.83 (5.78, 5.88)	-2.41 (-2.51, -2.3)	14,835(20.40)	1.31 (1.29, 1.33)	-1.71 (-2.12, -1.31)
**0 to 39**	437(0.60)	0.11 (0.1, 0.12)	-2.42 (-5.13, -0.09)	255(0.35)	0.06 (0.06, 0.07)	-1.48 (-5.24, 1.09)
**40 to 54**	8740(12.02)	4.46 (4.36, 4.55)	-2.78 (-3.96, -1.93)	2628(3.61)	1.34 (1.29, 1.4)	-0.88 (-2.76, 0.72)
**55 to 69**	27,048(37.20)	18.9 (18.67, 19.12)	-2.12 (-2.38, -1.97)	6799(9.35)	4.46 (4.36, 4.57)	-1.75 (-2.43, -1.23)
**70 to 84**	18,798(25.85)	28.1 (27.7, 28.51)	-2.07 (-2.43, -1.75)	4478(6.16)	5.24 (5.09, 5.4)	-1.97 (-2.74, -1.25)
**+ 85**	2859(3.93)	22.74 (21.91, 23.59)	-1.85 (-2.64, -1.05)	675(0.93)	2.6 (2.4, 2.8)	-0.21 (-3.07, 2.6)

**Abbreviations**: NHW: Non-Hispanic White; NHB: Non-Hispanic Black; ASIR: Age-standardized incidence rate; CI: Confidence interval, AAPC: Average annual percent change; N/A: Not available

#### Men

Over 2000–2019, a total of 79,740 cases of SCC were reported in men. The majority of cases were among NHWs (72.59%) and those aged 55–69 years (47.42%). Those in the 70–84 age group had the highest ASIR (27.94 [27.59, 28.29]). The largest decrease in ASIRs was in those between 40 and 54 years (AAPC: -3.80% [-4.77, -3.10]) (Table 3).

There were 8,053 (10.10%) cases among Hispanics. The majority of them aged 55–69 years (45.05%). The overall ASIR was 4.66 (4.55, 4.77). Cases between 70 and 84 years had the highest ASIR among age groups (27.18 [26.12, 28.28]). Hispanics experienced a significant drop in ASIRs with an AAPC of -2.96% (-3.50, -2.39) with those in the 40–54 age group exhibiting the most significant drop in ASIR compared to the other age groups (AAPC: -4.91% [-6.46, -3.41]) (Table 3).

NHBs accounted for 14.03% of the cases diagnosed with SCC in men. A majority of NHB cases aged 55–69 years (53.32%). The overall ASIR was 8.62 (8.45, 8.79) per 100,000, with the highest ASIR observed in 70–84 years (40.33 [38.82, 41.88]). The overall AAPC in this groups was − 3.31% (-3.65, -2.97) with those in the 40–54 age group having the greatest drop in ASIR (AAPC: -5.39% [-6.61, -4.35]) (Table 3).

The majority of NHW cases aged 55–69 years (46.73%). The overall ASIR per 100,000 population was 5.83 (5.78, 5.88), with the highest ASIR observed in cases aged 70-84 years (28.10 [27.70, 28.51]). The overall AAPC for NHWs was − 2.41 (-2.51, -2.30) representing a marked decline in ASIRs with those between 40 and 54 years having the greatest decline compared to other age groups (-2.78% [-3.96, -1.93]) (Table 3).

#### Women

Between 2000 and 2019, a total of 19,507 cases of SCC were reported in women. The majority of them were among NHWs (76.05%), and most of them aged 55–69 years (46.27%). Individuals aged 70–84 years had the largest ASIR (4.76 [4.64, 4.89]). Individuals < 39 years old had the greatest decline in ASIRs with an AAPC of -2.73% (-5.89, -0.48). (Table 3).

There were 1,359 (6.97%) cases among Hispanics and the majority of them aged 55–69 years (41.35%). The overall ASIR per 100,000 population for this group was 0.60 (0.57, 0.64). Cases among individuals 70–84 years had the highest ASIR (2.74 [2.46, 3.04]). There was a significant decrease in the ASIRs of Hispanic cases over the 2000–2019 period (AAPC: -3.62% [-4.41, -2.23]) with those between 55 and 69 years old experiencing the greatest fall in incidence rate (AAPC: -4.29% [-5.94, -0.80]) (Table 3).

NHBs constituted 14.82% of the cases of SCC in women. A significant portion of NHB cases aged 55–69 years (51.42%). The overall ASIR for NHB women was 1.63 (1.57, 1.69) with cases aged 55–69 years had the highest ASIR (6.08 [5.77, 6.39]). There was a significant decrease in ASIRs over 2000–2019 among NHB women (AAPC: -2.47% [-3.46, -1.49]) and those aged 40–54 years had the greatest decline in ASIRs (AAPC: -4.50% [-7.25, -2.07]) (Table 3).

Among women with SCC, there were 14,835 reported cases of NHWs. The majority of NHW cases were in the 55–69 age group (45.83%). The overall ASIR for NHW women was 1.31 (1.29, 1.33), with cases aged 70–84 years had the highest ASIR (5.24 [5.09, 5.40]). NHWs showed a decrease in ASIRs over 2000–2019 (AAPC: -1.71% [-2.12, -1.31]) and individuals aged 70–84 years had the largest decrease in ASIR from 2000 to 2019 (-1.97% [-2.74, -1.25]) (Table 3).

#### Age and sex patterns

The incident cases increased with age and peaked in 65–69 in women and 60–64 in men. In terms of the incidence rate, both sexes had a substantial increase in incidence rate after 35 years old, peaked in 75–79 for men and 70–74 for women. Men showed a higher incidence rate across all age groups (Figure S4).

### Neuroendocrine carcinoma

#### Overall incidence

Over the period spanning 2000 to 2019, there were a total of 545 cases of neuroendocrine carcinoma in all age groups. The majority of them were in men (64.22%), NHWs (75.05%), and individuals aged 55–69 years (48.78%). The ASIR per 100,000 population was 0.02 (0.02, 0.03) for men and 0.01 (0.01, 0.01) for women. There were no significant changes in ASIRs over 2000–2019 (Table 4, Figure S5, Figure S6, and Figure S7).

**Table 4:** Counts and age-standardized rate of neuroendocrine carcinoma incidence per 100,000 and average annual percent change from 2000 to 2019 in the United States, by age, sex, and race

**All race/ethnicities**						
**Age group (years)**	**Men**			**Women**		
	**Case (%)**	**ASIR (95% CI)**	**AAPC (95% CI)**	**Case (%)**	**ASIR (95% CI)**	**AAPC (95% CI)**
**All**	350(64.22)	0.02 (0.02, 0.03)	1.12 (-1.34, 3.98)	195(35.78)	0.01 (0.01, 0.01)	-0.32 (-3.26, 1.58)
**0 to 39**	13(2.39)	0 (0, 0)	N/A	9(1.65)	0 (0, 0)	N/A
**40 to 54**	61(11.19)	0.02 (0.02, 0.03)	N/A	39(7.16)	0.01 (0.01, 0.02)	N/A
**55 to 69**	170(31.19)	0.08 (0.07, 0.1)	-0.15 (-3.09, 3.46)	92(16.88)	0.04 (0.03, 0.05)	-1.18 (-5.15, 3.56)
**70 to 84**	92(16.88)	0.1 (0.08, 0.13)	2.58 (-1.06, 7.47)	46(8.44)	0.04 (0.03, 0.05)	N/A
**+ 85**	14(2.57)	0.09 (0.05, 0.15)	N/A	9(1.65)	0.03 (0.01, 0.05)	N/A
**Hispanic**						
**Age groups**	**Men**			**Women**		
	**Case (%)**	**ASIR (95% CI)**	**AAPC (95% CI)**	**Case (%)**	**ASIR (95% CI)**	**AAPC (95% CI)**
**All**	28(68.29)	0.01 (0.01, 0.02)	N/A	13(31.71)	0.01 (0, 0.01)	N/A
**0 to 39**	3(7.32)	0 (0, 0)	N/A	1(2.44)	0 (0, 0)	N/A
**40 to 54**	7(17.07)	0.01 (0, 0.02)	N/A	2(4.88)	0 (0, 0.01)	N/A
**55 to 69**	12(29.27)	0.05 (0.02, 0.08)	N/A	8(19.51)	0.03 (0.01, 0.05)	N/A
**70 to 84**	6(14.63)	0.06 (0.02, 0.14)	N/A	1(2.44)	0.01 (0, 0.04)	N/A
**+ 85**	0(0.00)	0 (0, 0.27)	N/A	1(2.44)	0.04 (0, 0.22)	N/A
**NHB**						
**Age groups**	**Men**			**Women**		
	**Case (%)**	**ASIR (95% CI)**	**AAPC (95% CI)**	**Case (%)**	**ASIR (95% CI)**	**AAPC (95% CI)**
**All**	45(60.00)	0.03 (0.02, 0.04)	N/A	30(40.00)	0.02 (0.01, 0.02)	N/A
**0 to 39**	1 (1.33)	0 (0, 0.01)	N/A	0 (0.00)	0 (0, 0)	N/A
**40 to 54**	6 (8.00)	0.02 (0.01, 0.04)	N/A	9 (12.00)	0.02 (0.01, 0.04)	N/A
**55 to 69**	28 (37.33)	0.14 (0.09, 0.21)	N/A	17 (22.67)	0.07 (0.04, 0.11)	N/A
**70 to 84**	10 (13.33)	0.15 (0.07, 0.28)	N/A	4 (5.33)	0.04 (0.01, 0.1)	N/A
**+ 85**	0 (0.00)	0 (0, 0.39)	N/A	0 (0.00)	0 (0, 0.15)	N/A
**NHW**						
**Age groups**	**Men**			**Women**		
	**Case (%)**	**ASIR (95% CI)**	**AAPC (95% CI)**	**Case (%)**	**ASIR (95% CI)**	**AAPC (95% CI)**
**All**	260(63.57)	0.03 (0.02, 0.03)	1.49 (-0.98, 4.42)	149(36.43)	0.01 (0.01, 0.02)	-0.62 (-3.89, 2.77)
**0 to 39**	8(1.96)	0 (0, 0)	N/A	8(1.96)	0 (0, 0)	N/A
**40 to 54**	46(11.25)	0.02 (0.02, 0.03)	N/A	28(6.85)	0.01 (0.01, 0.02)	N/A
**55 to 69**	123(30.07)	0.09 (0.07, 0.1)	1.13 (-2.66, 5.97)	66(16.14)	0.04 (0.03, 0.05)	-0.1 (-4.06, 4.7)
**70 to 84**	72(17.60)	0.11 (0.08, 0.14)	N/A	40(9.78)	0.05 (0.03, 0.06)	N/A
**+ 85**	11(2.69)	0.09 (0.04, 0.16)	N/A	7(1.71)	0.03 (0.01, 0.06)	N/A

**Abbreviations**: NHW: Non-Hispanic White; NHB: Non-Hispanic Black; ASIR: Age-standardized incidence rate; CI: Confidence interval, AAPC: Average annual percent change; N/A: Not available

#### Men

From 2000 to 2019, there were a total of 350 cases of neuroendocrine carcinoma reported in men. The majority of them were among NHWs (74.29%), with the highest occurrence in individuals aged 55–69 years (48.57%). None of the age groups had a significant change in the incidence rate over 2000–2019 (Table 4).

There were 28 (8.00%) cases among Hispanics. The majority of them aged 55–69 years (42.85%). The overall ASIR per 100,000 population for Hispanic men was 0.01 (0.01, 0.02). Cases aged 70–84 years had the highest ASIR among all age groups (0.06 [0.02, 0.14]). NHBs accounted for 12.86% of the cases of neuroendocrine carcinoma in men. A majority of NHB cases occurred in individuals aged 55–69 years (62.22%). The overall ASIR per 100,000 population for NHBs was 0.03 (0.02, 0.04). There were 260 reported neuroendocrine carcinoma cases among NHWs. The majority of NHW cases occurred in individuals aged 55–69 years (47.31%). The overall ASIR per 100,000 population for NHW men was 0.03 (0.02, 0.03) (Table 4).

#### Women

Between 2000 and 2019, a total of 195 (35.78%) cases of neuroendocrine carcinoma were reported in women. The majority of them were among NHWs (76.41%) and those aged 55–69 years (47.18%) (Table 4).

Among all reported cases, 13 (6.67%) were among Hispanics. The overall ASIR per 100,000 population for Hispanic women was 0.01 (0.00, 0.01). The majority of cases were individuals aged 55–69 years (66.67%), and cases over 85 had the highest ASIR (0.04 [0.00, 0.22]). NHBs constituted 17.95% of the cases of neuroendocrine carcinoma in women. Most NHB cases aged 55–69 year (48.57%). The overall ASIR per 100,000 population for NHB women was 0.02 (0.01, 0.02) with those in 55–69 age group showing the highest ASIR in comparison to the other age groups at 0.07 [0.04, 0.11]. Among women with neuroendocrine carcinoma, there were 149 reported cases of NHWs. Most cases among NHW individuals occurred in the 55–69 age bracket (44.30%). The ASIR per 100,000 population for NHW women was 0.01 (0.01–0.02). Moreover, those aged 70–84 had the highest ASIR at 0.05 (0.03–0.06) (Table 4).

#### Age and sex patterns

Both males and females showed an increase in number of incident cases after 35–39 years. It peaked in 60–64 for both sexes. Regarding the incidence rates, men and women displayed minimal to no fluctuation until the ages of 35–39 years. Then, men had an increase, peaking at 65–69 age group. Women had an increase until 60–64 with a peak in 60–74 years. There were not significant differences in incidence rate between males and females (Figure S8).

### Chondrosarcoma

#### Overall incidence

From 2000 to 2019, a total of 317 cases of chondrosarcoma across all age groups were reported in the US. The majority of them were men (73.19%), NHWs (86.44%), and individuals aged 55–69 years (41.01%). The ASIR per 100,000 population was 0.02 (0.01, 0.02) for men and 0.01 (0.00, 0.01) for women. NHW men had the highest ASIR (0.02 [0.02, 0.02]) (Table 5, Figure S9, Figure S10, and Figure S11).

**Table 5:** Counts and age-standardized rate of chondrosarcoma incidence per 100,000 and average annual percent change from 2000 to 2019 in the United States, by age, sex, and race

**All race/ethnicities**						
**Age group (years)**	**Men**			**Women**		
	**Case (%)**	**ASIR (95% CI)**	**AAPC (95% CI)**	**Case (%)**	**ASIR (95% CI)**	**AAPC (95% CI)**
**All**	232(73.19)	0.02 (0.01, 0.02)	-0.89 (-4, 2.5)	85(26.81)	0.01 (0, 0.01)	-0.8 (-5.52, 4.16)
**0 to 39**	11(3.47)	0 (0, 0)	N/A	5(1.58)	0 (0, 0)	N/A
**40 to 54**	49(15.46)	0.02 (0.01, 0.02)	N/A	18(5.68)	0.01 (0, 0.01)	N/A
**55 to 69**	97(30.60)	0.05 (0.04, 0.06)	-2.56 (-7.99, 3.21)	33(10.41)	0.01 (0.01, 0.02)	N/A
**70 to 84**	69(21.77)	0.08 (0.06, 0.1)	0.26 (-3.08, 4.11)	26(8.20)	0.02 (0.01, 0.03)	N/A
**+ 85**	6(1.89)	0.04 (0.01, 0.08)	N/A	3(0.95)	0.01 (0, 0.03)	N/A
**Hispanic**						
**Age groups**	**Men**			**Women**		
	**Case (%)**	**ASIR (95% CI)**	**AAPC (95% CI)**	**Case (%)**	**ASIR (95% CI)**	**AAPC (95% CI)**
**All**	19(73.08)	0.01 (0.01, 0.01)	N/A	7(26.92)	0 (0, 0.01)	N/A
**0 to 39**	3(11.54)	0 (0, 0)	N/A	2(7.69)	0 (0, 0)	N/A
**40 to 54**	8(30.77)	0.01 (0.01, 0.03)	N/A	2(7.69)	0 (0, 0.01)	N/A
**55 to 69**	4(15.38)	0.02 (0, 0.04)	N/A	2(7.69)	0.01 (0, 0.02)	N/A
**70 to 84**	4(15.38)	0.04 (0.01, 0.11)	N/A	1(3.85)	0.01 (0, 0.04)	N/A
**+ 85**	0(0.00)	0 (0, 0.27)	N/A	0(0.00)	0 (0, 0.14)	N/A
**NHB**						
**Age groups**	**Men**			**Women**		
	**Case (%)**	**ASIR (95% CI)**	**AAPC (95% CI)**	**Case (%)**	**ASIR (95% CI)**	**AAPC (95% CI)**
**All**	6(54.55)	0.01 (0, 0.01)	N/A	5(45.45)	0 (0, 0.01)	N/A
**0 to 39**	0(0.00)	0 (0, 0)	N/A	0(0.00)	0 (0, 0)	N/A
**40 to 54**	1(9.09)	0 (0, 0.02)	N/A	3(27.27)	0.01 (0, 0.02)	N/A
**55 to 69**	2(18.18)	0.01 (0, 0.04)	N/A	2(18.18)	0.01 (0, 0.03)	N/A
**70 to 84**	3(27.27)	0.05 (0.01, 0.14)	N/A	0(0.00)	0 (0, 0.03)	N/A
**+ 85**	0(0.00)	0 (0, 0.39)	N/A	0(0.00)	0 (0, 0.15)	N/A
**NHW**						
**Age groups**	**Men**			**Women**		
	**Case (%)**	**ASIR (95% CI)**	**AAPC (95% CI)**	**Case (%)**	**ASIR (95% CI)**	**AAPC (95% CI)**
**All**	202(73.72)	0.02 (0.02, 0.02)	-1.18 (-5.16, 2.9)	72(26.28)	0.01 (0.01, 0.01)	-1.78 (-7.47, 3.77)
**0 to 39**	8(2.92)	0 (0, 0)	N/A	3(1.09)	0 (0, 0)	N/A
**40 to 54**	38(13.87)	0.02 (0.01, 0.03)	N/A	13(4.74)	0.01 (0, 0.01)	N/A
**55 to 69**	90(32.85)	0.06 (0.05, 0.08)	-2.61 (-8.85, 3.84)	29(10.58)	0.02 (0.01, 0.03)	N/A
**70 to 84**	60(21.90)	0.09 (0.07, 0.12)	N/A	24(8.76)	0.03 (0.02, 0.04)	N/A
**+ 85**	6(2.19)	0.05 (0.02, 0.1)	N/A	3(1.09)	0.01 (0, 0.03)	N/A

**Abbreviations**: NHW: Non-Hispanic White; NHB: Non-Hispanic Black; ASIR: Age-standardized incidence rate; CI: Confidence interval, AAPC: Average annual percent change; N/A: Not available

#### Men

Between 2000 and 2019, a total of 232 cases of chondrosarcoma were reported in men. The majority of them were NHWs (87.07%) and aged 55–69 years (41.81%). Individuals between 70 and 84 years had the highest ASIR (0.08 [0.06, 0.10]). There was no significant change in the ASIRs among the age groups over 2000–2019 (Table 5).

There were 19 (8.19%) cases in Hispanics. The majority of them aged 40–54 years (42.11%). The overall ASIR per 100,000 population for Hispanic men was 0.01 (0.01, 0.01). NHBs accounted for 2.59% of the cases of chondrosarcoma in men. A majority of NHB cases were in individuals aged 70–84 years (50.00%). The overall ASIR per 100,000 population for NHB men was 0.01 (0.00, 0.01). There were 202 reported chondrosarcoma cases among NHWs. The majority of NHW cases aged 55–69 years (44.55%). The overall ASIR per 100,000 population for NHW men was 0.02 (0.02, 0.02) (Table 5).

#### Women

There were 85 reported cases of chondrosarcoma in women. The majority of these cases were NHW women, comprising 84.71%, and the largest age group affected was those aged 55–69 years, accounting for 38.82% of the cases. The highest ASIR was observed in women aged 70–84 years, with a rate of 0.02 (0.01, 0.03) (Table 5).

Among all reported cases, 8.24% were among Hispanics. NHBs constituted 5.88% of the cases of chondrosarcoma in women. Among women with chondrosarcoma, there were 72 reported cases of NHWs. The majority of NHW cases aged 55–69 years (40.28%) (Table 5).

#### Age and sex patterns

The highest incident number and rate for men was in 60–64 and 75–79 years. For women, the incident number and rate were largest in 70–74 years. There were not remarkable differences between males and females in incidence rates of chondrosarcoma (Figure S12).

## Discussion

This population-based study provided an extensive analysis of laryngeal cancer incidence trends in the US over a 21-year period spanning 2000–2020. Utilizing data from the SEER cancer registries representing nearly half the US population, we conducted a comprehensive assessment of temporal patterns stratified by key demographic and morphological subtypes, taking into account the rare laryngeal cancer entities, as well as the impact of the COVID-19 pandemic on the reported incidence rates. To the best of our knowledge, this is the first study to utilize the most recent iteration of SEER database to assess the changing trends of laryngeal cancer incidence, allowing for an accurate determination of epidemiological patterns.

Overall, our findings demonstrated a decreasing trend in incidence of laryngeal cancer across all races and ethnicities in both sexes within 2000–2019. The most prevalent subtype was SCC, followed by neuroendocrine carcinoma and laryngeal chondrosarcoma. The sex disparities in laryngeal cancer were evident, with men consistently exhibiting higher incidence rates across all age groups and a men’s predominance observed in all three morphological subtypes. Furthermore, the decrease in incidence rate was observed in all age groups among men. Additionally, in women, a decrease in AAPC was observed in all age groups, except for ages below 39 and above 85 years. Moreover, in both men and women, NHBs exhibited the highest incidence rate during the study period. Regarding AAPC, NHB men and Hispanic women demonstrated the greatest decrease in incidence rates across 2000–2019. The highest incidence rates across all ethnicities and sexes were attributed to NHB men. Narrowing our analysis to the recent period of 2015–2019, NHB men still remained as the group with higher ASIR, suggesting the high occurrence of laryngeal cancer among African-American men. We generally observed similar trends in laryngeal SCC, the most common subtype of laryngeal cancer. Accordingly, a decline in incidence rates of SCC was observed in both men and women of all races. In a similar fashion, a decreasing trend was observed for all races/ethnicities across 2000–2019. Analyzing the age patterns for each sex demonstrated the peak incidence rate in the 75–79 age group in men and 70–74 in women.

Our study’s findings of an overall decline in laryngeal cancer incidence align with preceding evidence, pointing to a reduction in rates within the country. Findings of a recent study on the trends of laryngeal cancer in the US within 1986–2018 demonstrated a 55% decrease in incidence rates, from 5.00 to 2.26 per 100,000 individuals during the study period, with declining rates in both men and women. However, no analysis was performed according to race and histological subtypes [8]. Results from a previous study using the SEER database reported a decrease in laryngeal cancer incidence by 1.9% from 2002 to 2012. Accordingly, men and women demonstrated a 2.09% and 1.71% decreasing rate, respectively. The most pronounced decline was observed in the 40–49 age group (APC: 3.82%) and 50–59 age group (APC: 2.16%) [7]. Overall, the decreasing rates of laryngeal cancer in the US have been attributed to decreased risk factors, particularly tobacco smoking [31]. Despite the decreasing rates, the racial disparities in the incidence rates, as observed in terms of higher rates among black races, have been attributed to racial differences in the association of head and neck cancers with tobacco and alcohol consumption [32]. However, there have also been disparities in the incidence rates according to socioeconomic status based on previous findings. For instance, in a study utilizing earlier versions of the SEER database, focusing on comparing the incidence trends of laryngeal cancer in urban and rural areas of the US, demonstrated that the adjusted incidence rates declined from 4.9 cases in 1975 to 2.5 per 100,000 people in 2015 among urban residents, and decreased from 3.0 in 1975 to 2.5 per 100,000 people in 2015 among rural residents. Accordingly, rural residents were found to have a decreasing incidence of 0.5% annually, while urban residents have had a much steeper decline in incidence of 2.6% annually since 1987, with significant differences in the incidence trends [33]. The disparities between low and high socioeconomic regions of the US were also observed in another study, where incidence rates of laryngeal cancer decreased in high socioeconomic regions, but remained stable in low socioeconomic regions [34]. Therefore, persistent racial and socioeconomic disparities underscore the need for targeted public health interventions and warrant more research into the changing patterns of risk factors.

Besides SCC as the most prevalent subtype of laryngeal cancer, we also analyzed the trends of two rare entities of laryngeal cancers, namely laryngeal neuroendocrine carcinoma and laryngeal chondrosarcoma. Our findings demonstrated that neuroendocrine carcinoma constituted only 0.52% of all laryngeal cancers within 2000–2019, with an ASIR of 0.02 in men and 0.01 per 100,000 in women, which is comparable to previous findings in the US. Accordingly, a previously published epidemiological trend of laryngeal neuroendocrine carcinoma within 1973–2011 in the US demonstrated an overall average incidence of 0.0117 per 100,000 population with a total of 257 cases, male predominance (62.6%) and a mean diagnosis age of 61.9 years [35]. Our findings demonstrated no notable changes among men and women for neuroendocrine carcinoma. Generally, neuroendocrine tumors of the larynx constitute an uncommon and morphologically heterogeneous type of cancer. Although laryngeal neuroendocrine carcinoma is a rare tumor and accounts for less than 1% of all laryngeal malignancies, the larynx is reported to be the most common location for these carcinomas [35]. These tumors represent the most prevalent non-squamous form of laryngeal cancers and are predominantly located in the supraglottic region [36]. These tumors are occasionally categorized as different types of typical carcinoid, atypical carcinoid, paraganglioma, and small cell neuroendocrine carcinoma, each with distinct biological factor, behavioral patterns, and survival rates [36].

Our analysis of laryngeal chondrosarcoma, another less prevalent type of laryngeal cancer, demonstrated that this entity accounts for only 0.30% of all laryngeal cancer within 2000–2019. Our findings revealed an ASIR of 0.02 in men and 0.01 per 100,000 in women. Unlike SCC, we did not observe any notable changes in the ASIRs of laryngeal chondrosarcoma in either of the sexes between 2000 and 2019. Our findings revealed a bimodal pattern of chondrosarcoma incidence rates in males and females. Findings from a previous study on the SEER data between 1973 and 2010 reported a total of 143 laryngeal chondrosarcoma, accounting for 0.2% of all laryngeal tumors [37]. The findings of that study also reported a significantly better survival for laryngeal chondrosarcoma compared to other laryngeal tumors [37]. Generally, laryngeal chondrosarcoma is an uncommon entity of laryngeal cancer originating from hyaline cartilage, with reports indicating that more than 56% of cases arise from cricoid cartilage [38]. A systematic review of 592 laryngeal chondrosarcoma cases has reported a 3:1 male-to-female ratio, and a 91.4% five-year disease-specific survival rate [38], which is comparable to an 88.6% five-year survival rate reported by a previous study among US cases [37].

Besides the rare entities of laryngeal tumors investigated in our study, several studies have previously reported the epidemiological aspects of other rare entities in the US. Findings from a study on laryngeal spindle cell carcinoma, a rare variant of laryngeal SCC, reported an incidence rate of 0.023 per 100,000 through 2000–2011 with APC of -0.12%. In this variant, a 7:1 male predominance was observed, with the majority of cases presenting in white races [13]. Findings from another study on a rare distinct entity of laryngeal cancer from typical SCC, namely laryngeal papillary SCC over 1973–2011 in the US, demonstrated that this histopathological subtype corresponds to 0.5% of all laryngeal tumors, with a 3:1 male predominance and no racial preference; however, the incidence trends were not investigated [12]. Reports from another rare variant of laryngeal cancer, laryngeal adenocarcinoma not otherwise specified, which refers to variants of minor salivary gland tumors of the larynx that do not align with other established histological subtypes, demonstrated an overall incidence of 0.008 per 100,000 within 2000–2012, with males (80.2%) and whites (84.7%) constituting the majority of cases [14].

To assess the influence of the COVID-19 pandemic on the documented cases of laryngeal cancer in the US, we scrutinized the data for 2020, evaluating the changes in the delayed ASIR in comparison to 2019. Results indicated a significant decrease in the incidence rate of laryngeal cancer across all racial groups and sexes subsequent to the onset of the pandemic. The most pronounced changes were observed among women, particularly Hispanic women. The COVID-19 pandemic in late 2019, resulted in reductions in non-COVID medical care including cancer screenings and patient referrals. Findings from a recent study on changes in cancer diagnosis in the first year of the COVID-19 pandemic in the US demonstrated that following the initiation of the COVID-19 pandemic, there was a marked decline in the monthly count of new cancer diagnoses across all stages. However, by the conclusion of 2020, the monthly counts rebounded, approaching levels comparable to the pre-pandemic period [39]. This observed pattern was evident across most cancer types, although it was most pronounced among Hispanic individuals. Interestingly, the patterns of underdiagnoses and stage distribution exhibited variability depending on the cancer site. Cancers for which asymptomatic screening services are recommended or those detectable through early signs and symptoms, experienced a notable decline in both the number and proportion of early-stage diagnoses during the initial 2–3 months of the pandemic. However, no data regarding the incidence changes of laryngeal cancer were reported [39]. Several other studies have also demonstrated changes in incidence rates of cancer after the outbreak of the pandemic, most possibly due to disrupted cancer screening and diagnosis [40–42]. Our findings revealed a remarkable decrease in ASIR of laryngeal cancer, a result which is in accordance with what observed among most other cancers.

Our study has several strengths and limitations. This is the first study leveraging the updated SEER database, incorporating delayed ASIRs for a more robust evaluation of laryngeal cancer incidence trends in the US. In addition to examining the overall laryngeal cancer incidence patterns, subgroup analyses encompassing distinct pathological subtypes were performed. Furthermore, recognizing the significant influence of the COVID-19 pandemic on cancer epidemiology, we analyzed shifts in laryngeal cancer incidence rates during the initial year of the pandemic compared to the preceding year, considering variations across races, age groups, and sexes. Nevertheless, it is crucial to acknowledge that our findings may be subject to the limitations inherent in the SEER database. The absence of documentation for lifestyle risk factors in the SEER database, and the unavailability of human papilloma virus infection status, which is found to be associated with laryngeal SCC [43], represent a limitation to assess the trends of potential risk factors. Moreover, the variability in data sources within the SEER database introduces the possibility of inaccuracies in the classification of demographic data, particularly race and ethnicity. We also did not report the incidence rates for some minor races/ethnicities like American Indian/Alaska Native and Native Hawaiian in our analysis since they had a very small sample size. In addition, although we performed delay adjustment for the calculation of the incidence rates for laryngeal cancer, there might be still the possibility of underestimation of the rates, particularly within the recent years of the study period, which could lead to bias.

## Conclusions

The analysis of laryngeal cancer incidence trends within 2000–2019 demonstrated a decline in incidence rates, with the most significant decline observed in NHBs. Moreover, in both men and women, NHB men exhibited the highest incidence rate during the study period. Also, a noteworthy decrease in the incidence rates was observed in all races and sexes following the COVID-19 pandemic in 2020. Further studies can investigate the risk factors of laryngeal cancer and the incidence rates attributable to each one.

### Electronic supplementary material

Below is the link to the electronic supplementary material.

Supplementary Material 1

## Data Availability

The data used in this study are available from the Surveillance, Epidemiology, and End Results Program (SEER) database.

## Abbreviations

NHW: Non-Hispanic White
NHB: Non-Hispanic Black
ASIR: Age-standardized incidence rate
CI: Confidence interval
AAPC: Average annual percent change
N/A: Not available
