# Supplementary material for: Laryngeal cancer incidence trends in the United States over 2000–2020: a population-based analysis
Source: Arch Public Health. 2024 Jul 10;82:106. doi: 10.1186/s13690-024-01333-1 (PMC11234729; doi:10.1186/s13690-024-01333-1)
Supplement: Supplementary file 1 — Supplementary Material 1 [file 13690_2024_1333_MOESM1_ESM.docx]

**Table S1.** Counts and age-standardized rate of laryngeal cancer incidence per 100,000 and average annual percent change from 2015 to 2019 in the United States, by age, sex, and race.

| **All race/ethnicities** | | | | | | |
| --- | --- | --- | --- | --- | --- | --- |
| **Age group (years)** | **Men** | | | **Women** | | |
|  | **Case (%)** | **ASIR (95% CI)** | **AAPC (95% CI)** | **Case (%)** | **ASIR (95% CI)** | **AAPC (95% CI)** |
| **All** | 20587 (80.03) | 4.92 (4.85, 4.99) | -2.77 (-2.94, -2.67) | 5138 (19.97) | 1.05 (1.03, 1.08) | -2.26 (-2.6, -1.93) |
| **0 to 39** | 164 (0.64) | 0.08 (0.07, 0.1) | -2.88 (-5.19, -0.79) | 105(0.41 | 0.05 (0.04, 0.06) | 9.49 (-3.66, 25.53) |
| **40 to 54** | 2417 (9.40) | 3.04 (2.92, 3.16) | -4.7 (-9.34, -3.8) | 771 (3.00) | 0.95 (0.88, 1.01) | -4.53 (-14.29, -2.48) |
| **55 to 69** | 10219 (39.72) | 16.01 (15.7, 16.32) | -0.85 (-2.19, -0.05) | 2561 (9.96) | 3.69 (3.54, 3.83) | 1.39 (-1.28, 3.26) |
| **70 to 84** | 6655 (25.87) | 25.08 (24.48, 25.69) | -3.41 (-4.72, -2.61) | 1441 (5.60) | 4.3 (4.08, 4.53) | -3.32 (-6.47, -2.35) |
| **+85** | 1132 (4.40) | 22.36 (21.07, 23.7) | -1.85 (-2.36, -1.32) | 260 (1.01) | 2.77 (2.45, 3.13) | -7.42 (-16.5, -1.51) |
| **Hispanic** | | | | | | |
| **Age groups** | **Men** | | | **Women** | | |
|  | **Case (%)** | **ASIR (95% CI)** | **AAPC (95% CI)** | **Case (%)** | **ASIR (95% CI)** | **AAPC (95% CI)** |
| **All** | 2404 (84.71) | 4.11 (3.94, 4.29) | -2.9 (-3.45, -2.31) | 434 (15.29) | 0.57 (0.52, 0.63) | -1.45 (-2.15, 0.07) |
| **0 to 39** | 36 (1.27) | 0.06 (0.04, 0.09) | -1.42 (-4.64, 1.99) | 23 (0.81) | 0.04 (0.03, 0.06) | -2.65 (-5.48, 0.16) |
| **40 to 54** | 315 (11.10) | 1.71 (1.53, 1.91) | -4.82 (-6.3, -3.36) | 82 (2.89) | 0.45 (0.36, 0.56) | -3.52 (-5.71, -1.21) |
| **55 to 69** | 1137 (40.06) | 11.72 (11.04, 12.43) | -3.19 (-3.98, -2.32) | 196 (6.91) | 1.8 (1.56, 2.07) | -0.65 (-2.47, 6.91) |
| **70 to 84** | 787 (27.73) | 24.99 (23.26, 26.82) | -2.31 (-3.38, -1.12) | 101 (3.56) | 2.35 (1.91, 2.86) | -2.49 (-3.87, -0.97) |
| **+85** | 129 (4.55) | 23.7 (19.78, 28.16) | -2.36 (-4.09, -0.2) | 32 (1.13) | 3.31 (2.27, 4.68) | N/A |
| **NHB** | | | | | | |
| **Age groups** | **Men** | | | **Women** | | |
|  | **Case (%)** | **ASIR (95% CI)** | **AAPC (95% CI)** | **Case (%)** | **ASIR (95% CI)** | **AAPC (95% CI)** |
| **All** | 2867 (78.70) | 7.22 (6.94, 7.51) | -3.26 (-3.6, -2.92) | 776 (21.30) | 1.44 (1.34, 1.55) | -2.49 (-3.46, -1.52) |
| **0 to 39** | 26 (0.71) | 0.1 (0.07, 0.15) | N/A | 11 (0.30) | 0.05 (0.02, 0.08) | N/A |
| **40 to 54** | 391 (10.73) | 4.33 (3.91, 4.79) | -5.39 (-6.62, -4.35) | 123 (3.38) | 1.2 (1, 1.43) | -4.58 (-7.13, -2.34) |
| **55 to 69** | 1596 (43.81) | 25.16 (23.93, 26.43) | -3.51 (-4.09, -2.91) | 432 (11.86) | 5.49 (4.98, 6.03) | -2.06 (-3.11, -0.91) |
| **70 to 84** | 769 (21.11) | 35.89 (33.37, 38.55) | -6.69 (-9.37, -4.05) | 184 (5.05) | 5.69 (4.9, 6.58) | -1.76 (-3.2, -0.29) |
| **+85** | 85 (2.33) | 26.84 (21.44, 33.18) | -1.61 (-4.26, 1.51) | 26 (0.71) | 3.51 (2.3, 5.15) | 0.74 (-2.45, 4.78) |
| **NHW** | | | | | | |
| **Age groups** | **Men** | | | **Women** | | |
|  | **Case (%)** | **ASIR (95% CI)** | **AAPC (95% CI)** | **Case (%)** | **ASIR (95% CI)** | **AAPC (95% CI)** |
| **All** | 14443 (79.23) | 5.18 (5.09, 5.26) | -2.51 (-2.69, -2.39) | 3786 (20.77) | 1.23 (1.19, 1.28) | -1.76 (-2.14, -1.39) |
| **0 to 39** | 95 (0.52) | 0.1 (0.08, 0.12) | -2.69 (-5.47, -0.35) | 68 (0.37) | 0.07 (0.05, 0.09) | 14.69 (0.38, 32.21) |
| **40 to 54** | 1616 (8.86) | 3.66 (3.48, 3.84) | -3.93 (-10.02, -2.79) | 554 (3.04) | 1.25 (1.15, 1.36) | -3.12 (-10.68, -1.26) |
| **55 to 69** | 7070 (38.78) | 16.77 (16.38, 17.17) | 0.15 (-1.23, 1.03) | 1876 (10.29) | 4.25 (4.06, 4.44) | 1.18 (-1.21, 4.09) |
| **70 to 84** | 4801 (26.32) | 25.29 (24.57, 26.02) | -3.16 (-5.71, -2.16) | 1101 (6.04) | 4.77 (4.49, 5.06) | -3.38 (-6.79, -2.28) |
| **+85** | 861 (4.72) | 22.82 (21.32, 24.39) | -1.69 (-2.44, -0.91) | 187 (1.03) | 2.69 (2.32, 3.11) | -3.24 (-10.46, -1.33) |

**Abbreviations:** NHW: Non-Hispanic White; NHB: Non-Hispanic Black; ASIR: Age-standardized incidence rate; CI: Confidence interval, AAPC: Average annual percent change; N/A: Not available.

**Table S2**. Results of the tests of parallelism for laryngeal cancer incidence rate over 2000-2019 in the United States.

| **Race/ethnicity** | **Sex** | **Subtypes** | **Race/ ethnicities** | **Sex** | **Subtype** | **P value** |
| --- | --- | --- | --- | --- | --- | --- |
| **Cohort 1** | | | **Cohort 2** | | |  |
| All | Female | Chondrosarcoma | All | Male | Chondrosarcoma | 0.97 |
| All | Female | Chondrosarcoma | All | Both | Chondrosarcoma | 0.15 |
| All | Male | Chondrosarcoma | All | Both | Chondrosarcoma | 0.23 |
| NHW | Female | Chondrosarcoma | NHW | Male | Chondrosarcoma | 0.79 |
| NHW | Female | Chondrosarcoma | NHW | Both | Chondrosarcoma | 0.79 |
| NHW | Male | Chondrosarcoma | NHW | Both | Chondrosarcoma | 0.75 |
| All | Female | All | All | Male | All | 0.08 |
| All | Female | All | All | Both | All | 0.17 |
| NHB | Female | All | NHB | Male | All | 0.07 |
| All | Female | NEC | All | Male | NEC | 0.07 |
| All | Female | NEC | All | Both | NEC | 0.07 |
| NHW | Female | NEC | NHW | Male | NEC | 0.21 |
| NHW | Female | NEC | NHW | Both | NEC | 0.21 |
| NHW | Male | NEC | NHW | Both | NEC | 0.12 |
| All | Female | SCC | All | Male | SCC | 0.08 |
| All | Female | SCC | All | Both | SCC | 0.06 |
| Hispanic | Male | SCC | Hispanic | Both | SCC | 0.06 |
| NHB | Female | SCC | NHB | Male | SCC | 0.07 |
| NHB | Female | SCC | NHB | Both | SCC | 0.05 |
| All | Female | Chondrosarcoma | NHW | Female | Chondrosarcoma | 0.39 |
| All | Male | Chondrosarcoma | NHW | Male | Chondrosarcoma | 0.52 |
| All | Both | Chondrosarcoma | NHW | Both | Chondrosarcoma | 0.75 |
| All | Female | All | Hispanic | Female | All | 0.09 |
| All | Female | All | NHB | Female | All | 0.52 |
| Hispanic | Female | All | NHB | Female | All | 0.13 |
| Hispanic | Female | All | NHW | Female | All | 0.28 |
| NHB | Female | All | NHW | Female | All | 0.14 |
| All | Male | All | Hispanic | Male | All | 0.06 |
| Hispanic | Male | All | NHB | Male | All | 0.06 |
| All | Both | All | Hispanic | Both | All | 0.14 |
| All | Female | NEC | NHW | Female | NEC | 0.11 |
| All | Male | NEC | NHW | Male | NEC | 0.39 |
| All | Both | NEC | NHW | Both | NEC | 0.15 |
| All | Female | SCC | Hispanic | Female | SCC | 0.06 |
| All | Female | SCC | NHB | Female | SCC | 0.5 |
| Hispanic | Female | SCC | NHB | Female | SCC | 0.13 |
| Hispanic | Female | SCC | NHW | Female | SCC | 0.25 |
| NHB | Female | SCC | NHW | Female | SCC | 0.14 |
| All | Male | SCC | Hispanic | Male | SCC | 0.08 |
| Hispanic | Male | SCC | NHB | Male | SCC | 0.06 |
| All | Both | SCC | Hispanic | Both | SCC | 0.15 |
| Hispanic | Both | SCC | NHB | Both | SCC | 0.11 |
| All | Female | Chondrosarcoma | All | Female | All | 0.29 |
| All | Female | Chondrosarcoma | All | Female | NEC | 0.75 |
| All | Female | Chondrosarcoma | All | Female | SCC | 0.33 |
| All | Female | All | All | Female | NEC | 0.32 |
| All | Female | All | All | Female | SCC | 0.35 |
| All | Female | NEC | All | Female | SCC | 0.33 |
| All | Male | Chondrosarcoma | All | Male | All | 0.08 |
| All | Male | Chondrosarcoma | All | Male | NEC | 0.13 |
| All | Male | Chondrosarcoma | All | Male | SCC | 0.12 |
| All | Male | All | All | Male | SCC | 0.38 |
| All | Both | Chondrosarcoma | All | Both | All | 0.07 |
| All | Both | Chondrosarcoma | All | Both | NEC | 0.3 |
| All | Both | All | All | Both | NEC | 0.06 |
| All | Both | All | All | Both | SCC | 0.27 |
| All | Both | NEC | All | Both | SCC | 0.05 |
| Hispanic | Female | All | Hispanic | Female | SCC | 0.51 |
| Hispanic | Male | All | Hispanic | Male | SCC | 0.11 |
| Hispanic | Both | All | Hispanic | Both | SCC | 0.12 |
| NHB | Female | All | NHB | Female | SCC | 0.83 |
| NHB | Male | All | NHB | Male | SCC | 0.52 |
| NHB | Both | All | NHB | Both | NEC | 0.1 |
| NHB | Both | All | NHB | Both | SCC | 0.63 |
| NHB | Both | NEC | NHB | Both | SCC | 0.15 |
| NHW | Female | Chondrosarcoma | NHW | Female | All | 0.99 |
| NHW | Female | Chondrosarcoma | NHW | Female | NEC | 0.57 |
| NHW | Female | Chondrosarcoma | NHW | Female | SCC | 0.96 |
| NHW | Female | All | NHW | Female | NEC | 0.1 |
| NHW | Female | All | NHW | Female | SCC | 0.11 |
| NHW | Female | NEC | NHW | Female | SCC | 0.16 |
| NHW | Male | Chondrosarcoma | NHW | Male | All | 0.31 |
| NHW | Male | Chondrosarcoma | NHW | Male | NEC | 0.09 |
| NHW | Male | Chondrosarcoma | NHW | Male | SCC | 0.39 |
| NHW | Male | All | NHW | Male | SCC | 0.13 |
| NHW | Both | Chondrosarcoma | NHW | Both | All | 0.16 |
| NHW | Both | Chondrosarcoma | NHW | Both | SCC | 0.21 |
| NHW | Both | All | NHW | Both | SCC | 0.21 |

**Abbreviations**: NHW: Non-Hispanic White; NHB: Non-Hispanic Black; NEC: Neuroendocrine carcinoma; SCC: Squamous Cell Carcinoma.


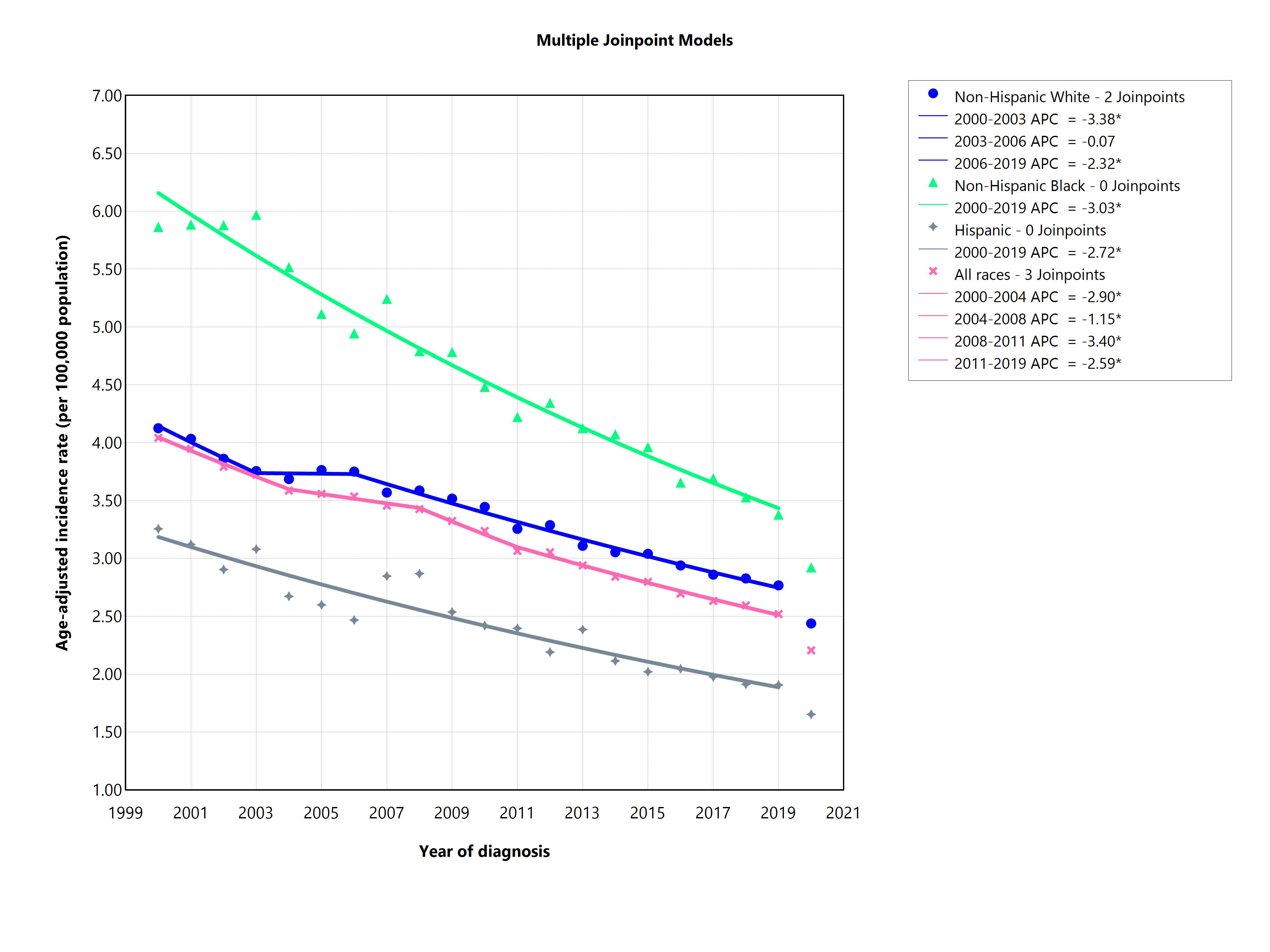


**Figure S1.** Delayed age-adjusted incidence rate of squamous cell carcinoma over 2000-2019 and in 2020 in the United States, by race. APC: annual percent change. * Represent p-value less than 0.05.


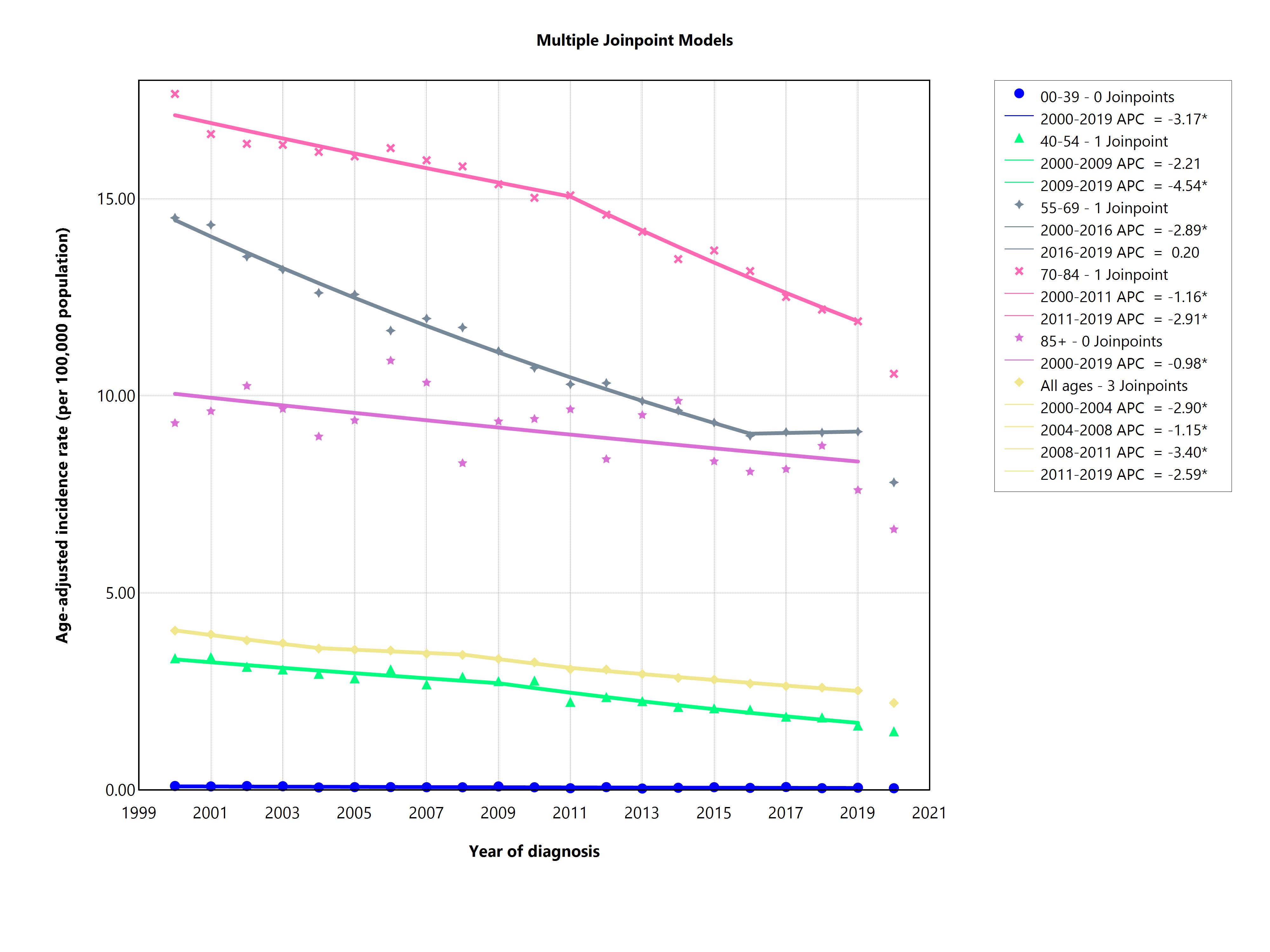


**Figure S2.** Delayed age-adjusted incidence rate of squamous cell carcinoma over 2000-2019 and in 2020 in the United States, by age. APC: annual percent change. * Represent p-value less than 0.05.


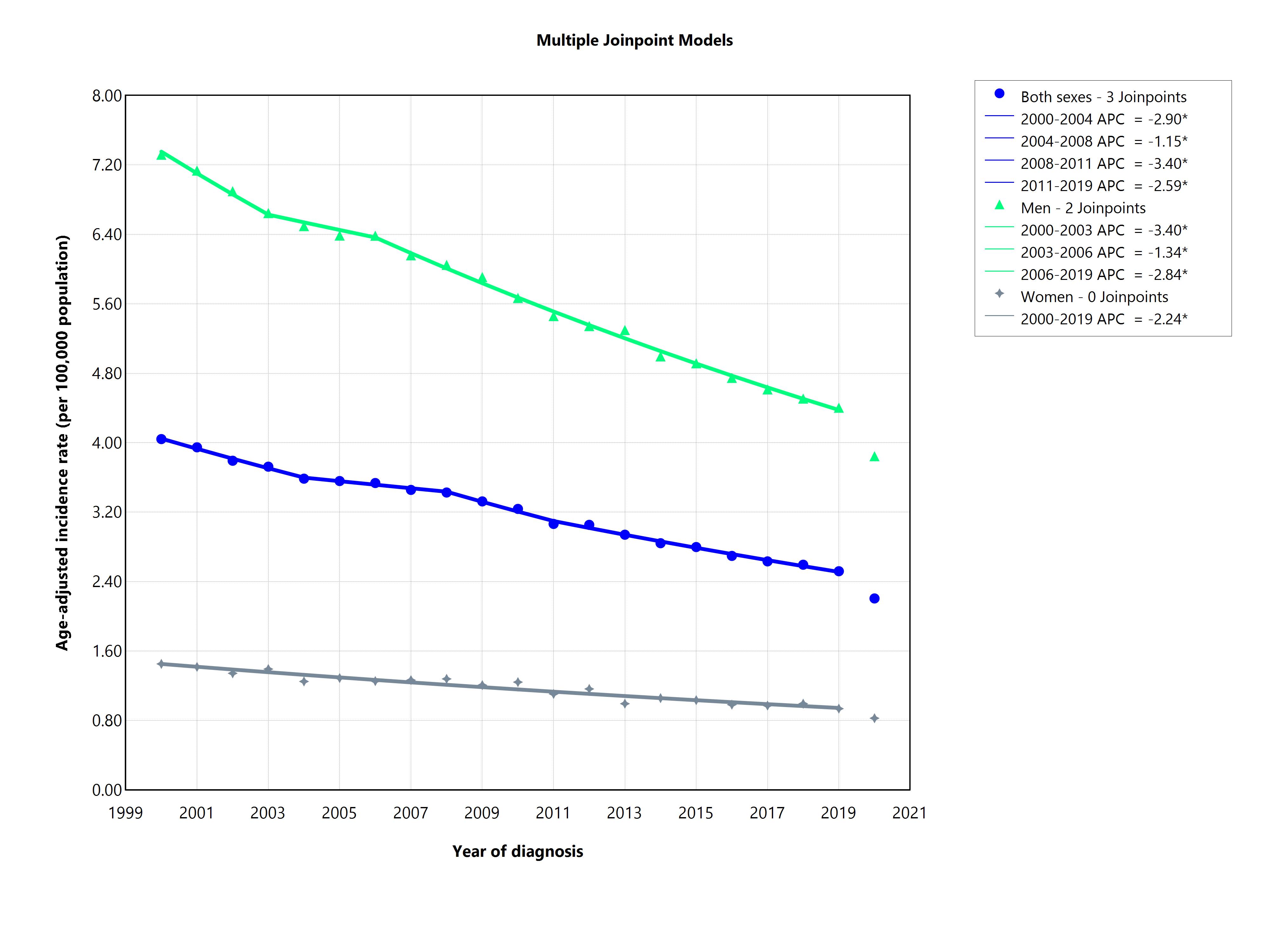


**Figure S3.** Delayed age-adjusted incidence rate of squamous cell carcinoma over 2000-2019 and in 2020 in the United States, by sex. APC: annual percent change. * Represent p-value less than 0.05.


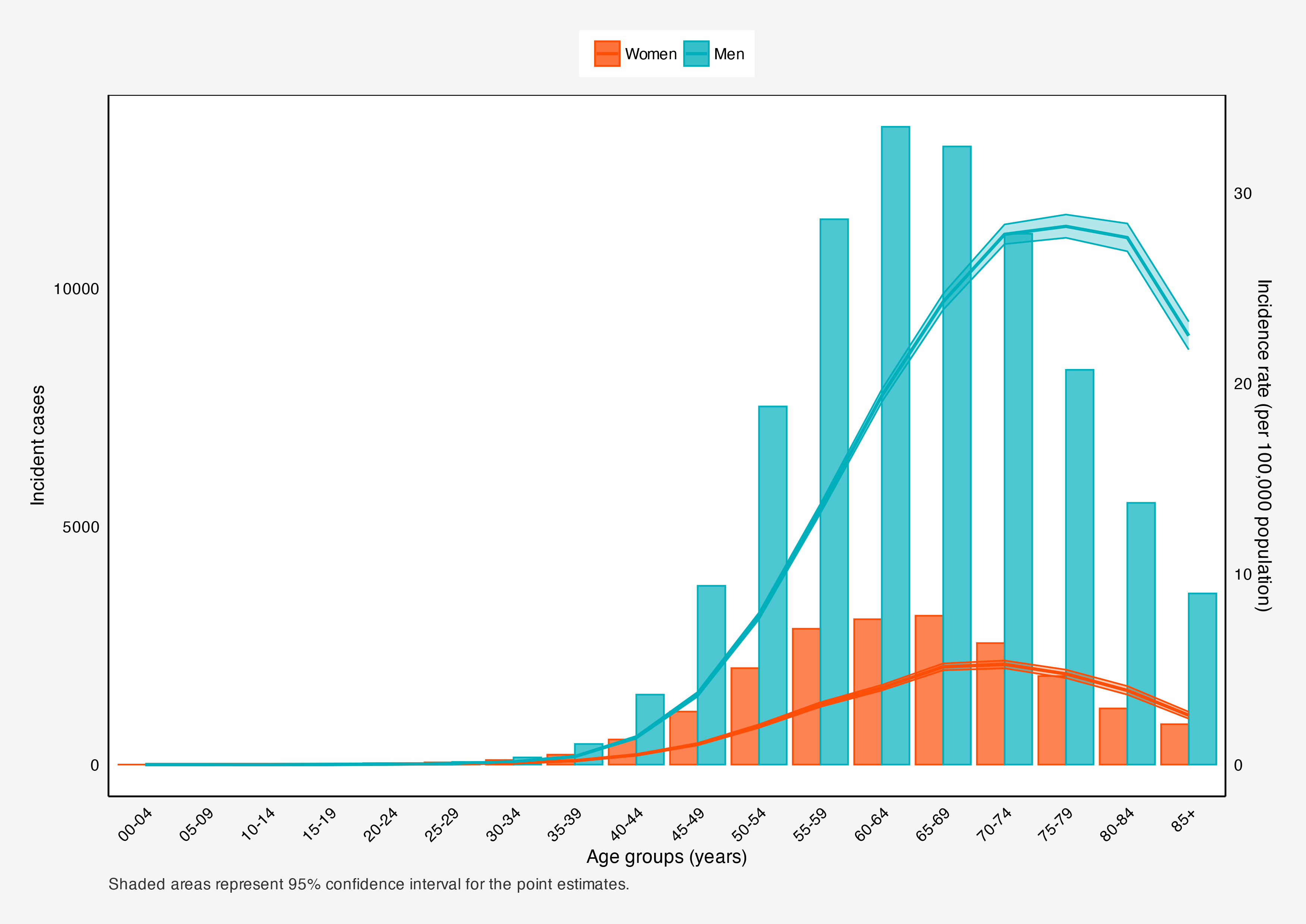


**Figure S4.** Incident numbers and incidence rate of squamous cell carcinoma in the United States among males and females in each age group. Shaded areas are the confidence interval range for the point estimates.


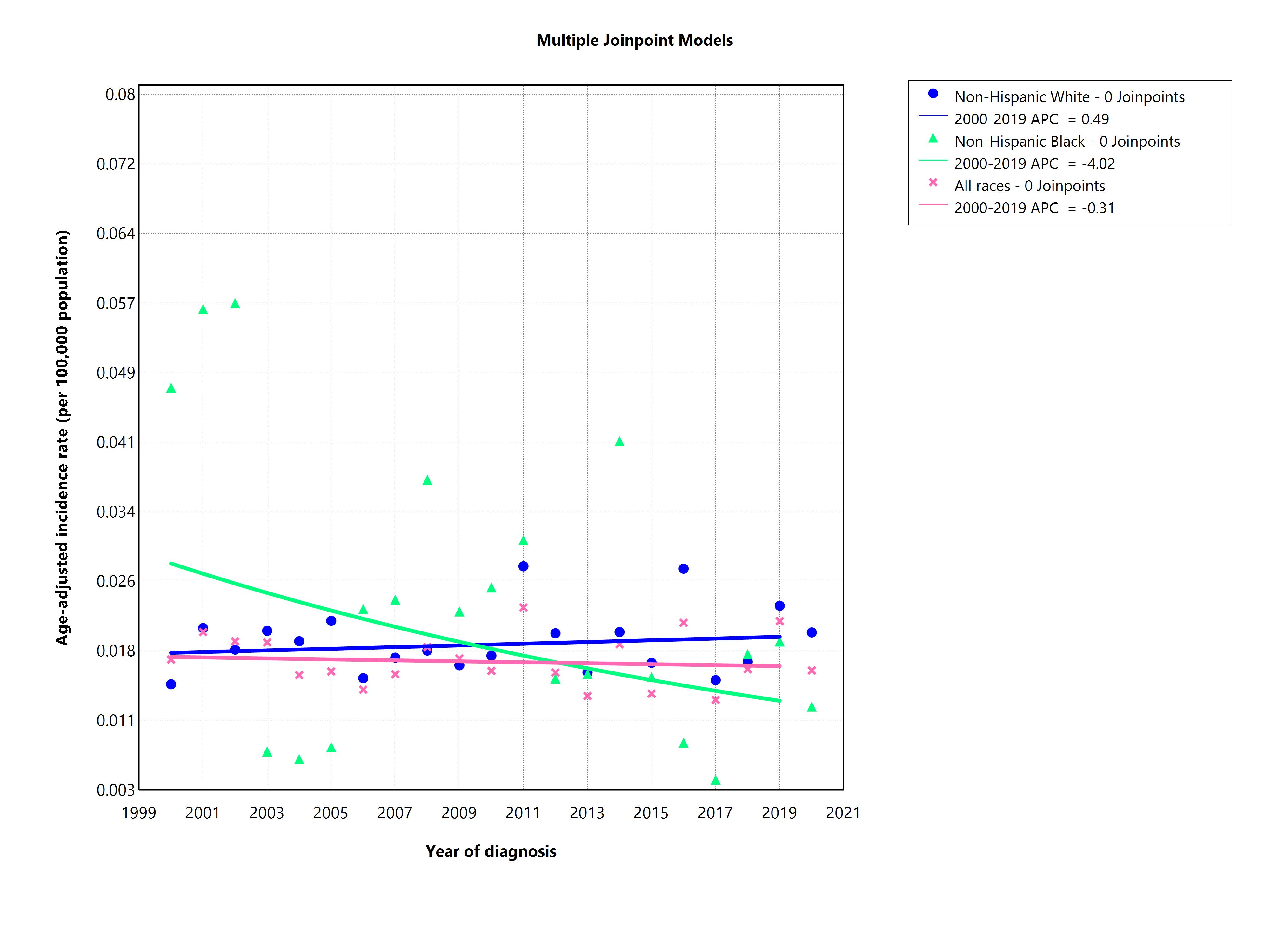


**Figure S5.** Delayed age-adjusted incidence rate of neuroendocrine carcinoma over 2000-2019 and in 2020 in the United States, by race. APC: annual percent change.


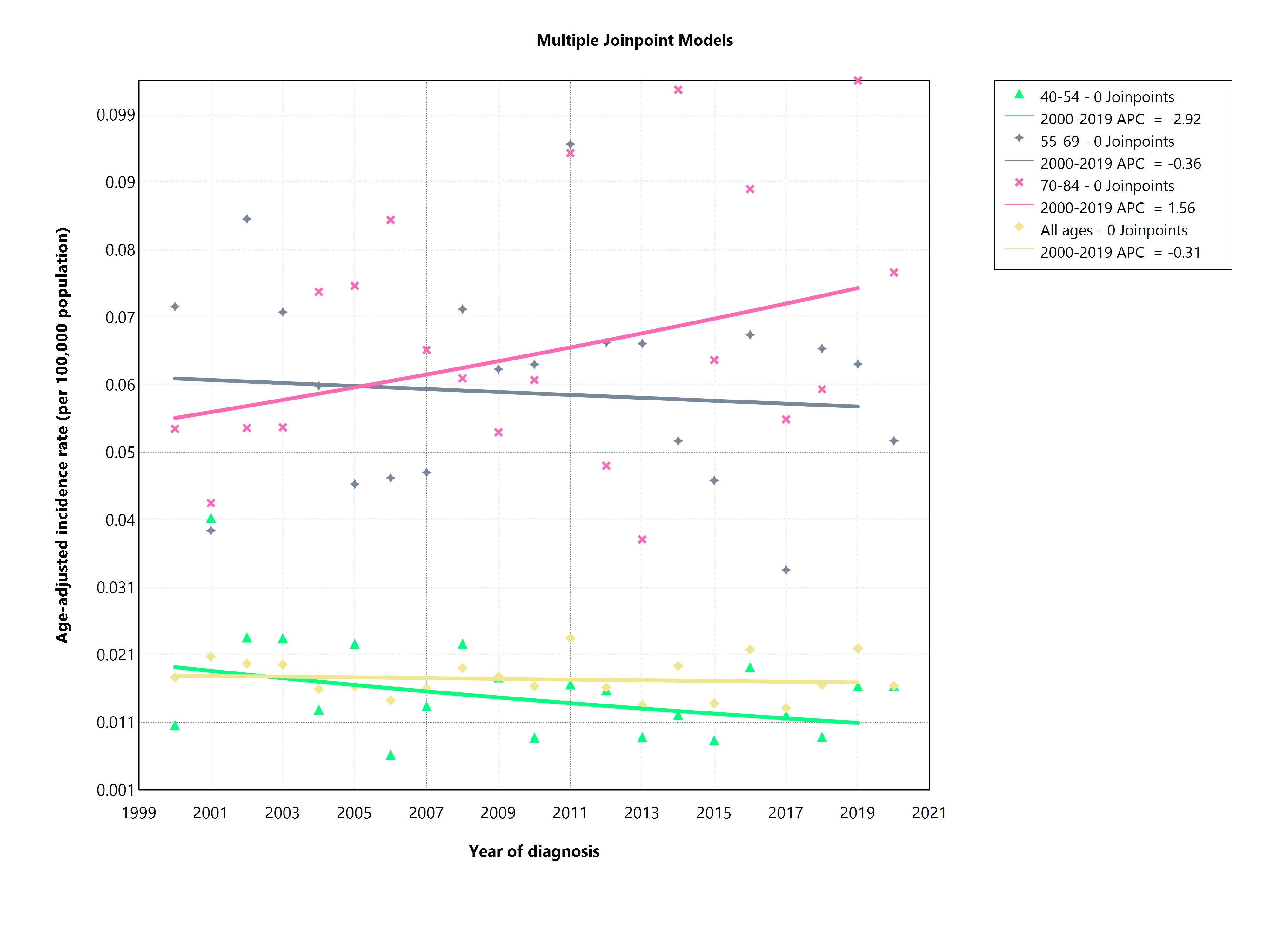


**Figure S6.** Delayed age-adjusted incidence rate of neuroendocrine carcinoma over 2000-2019 and in 2020 in the United States, by age. APC: annual percent change.


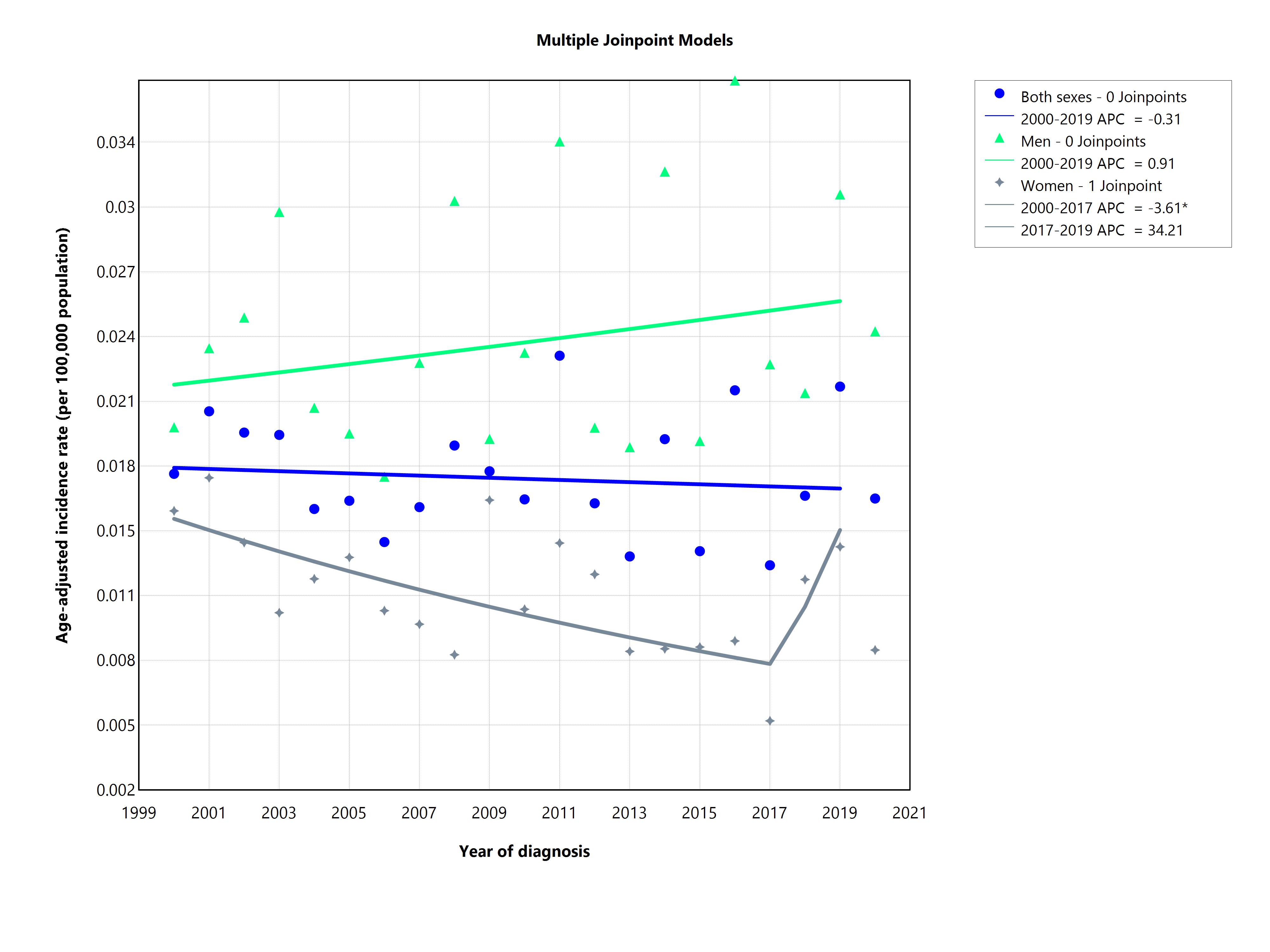


**Figure S7.** Delayed age-adjusted incidence rate of neuroendocrine carcinoma over 2000-2019 and in 2020 in the United States, by sex. APC: annual percent change. * Represent p-value less than 0.05.


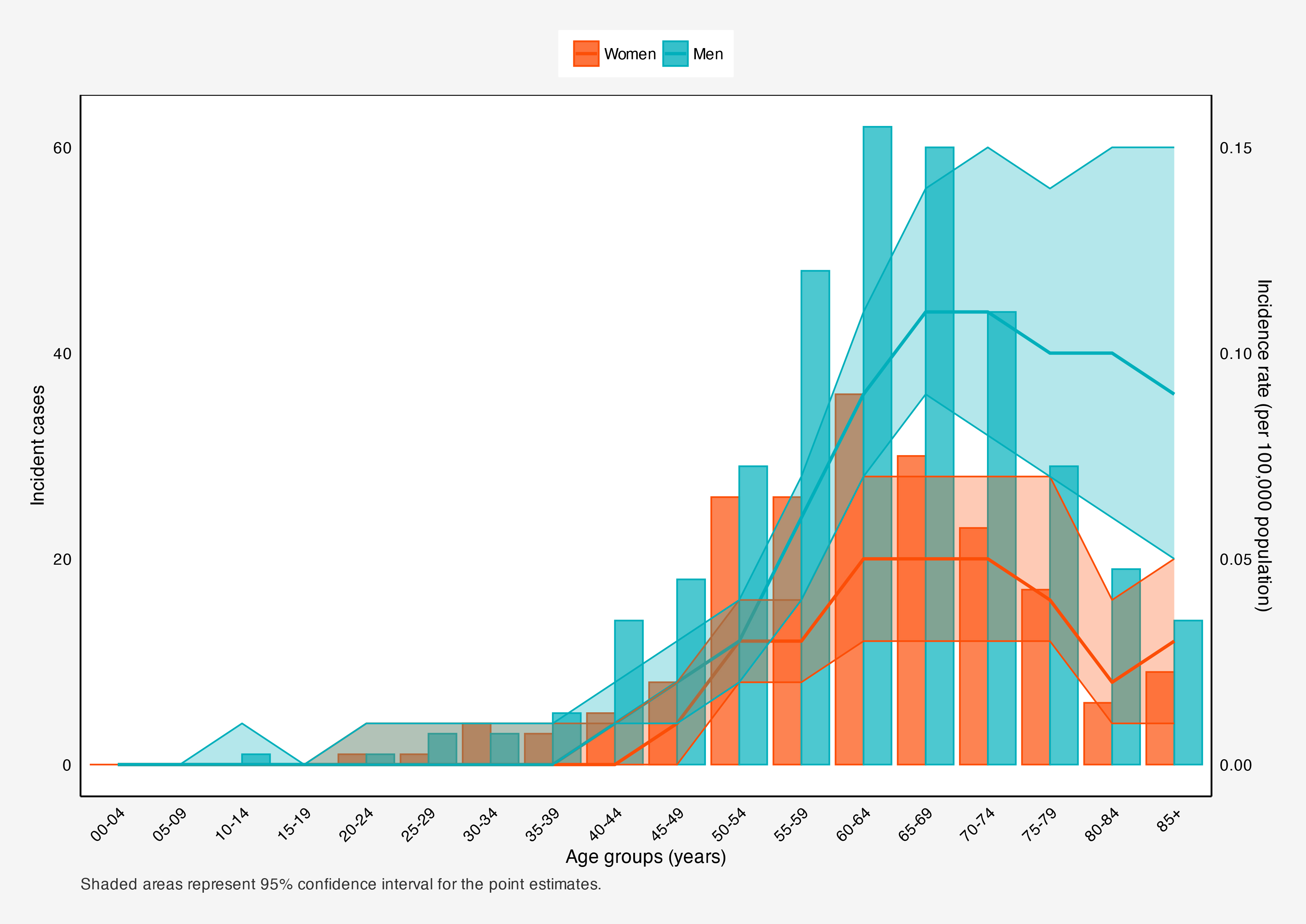


**Figure S8.** Incident numbers and incidence rate of neuroendocrine carcinoma in the United States among males and females in each age group. Shaded areas are the confidence interval range for the point estimates.


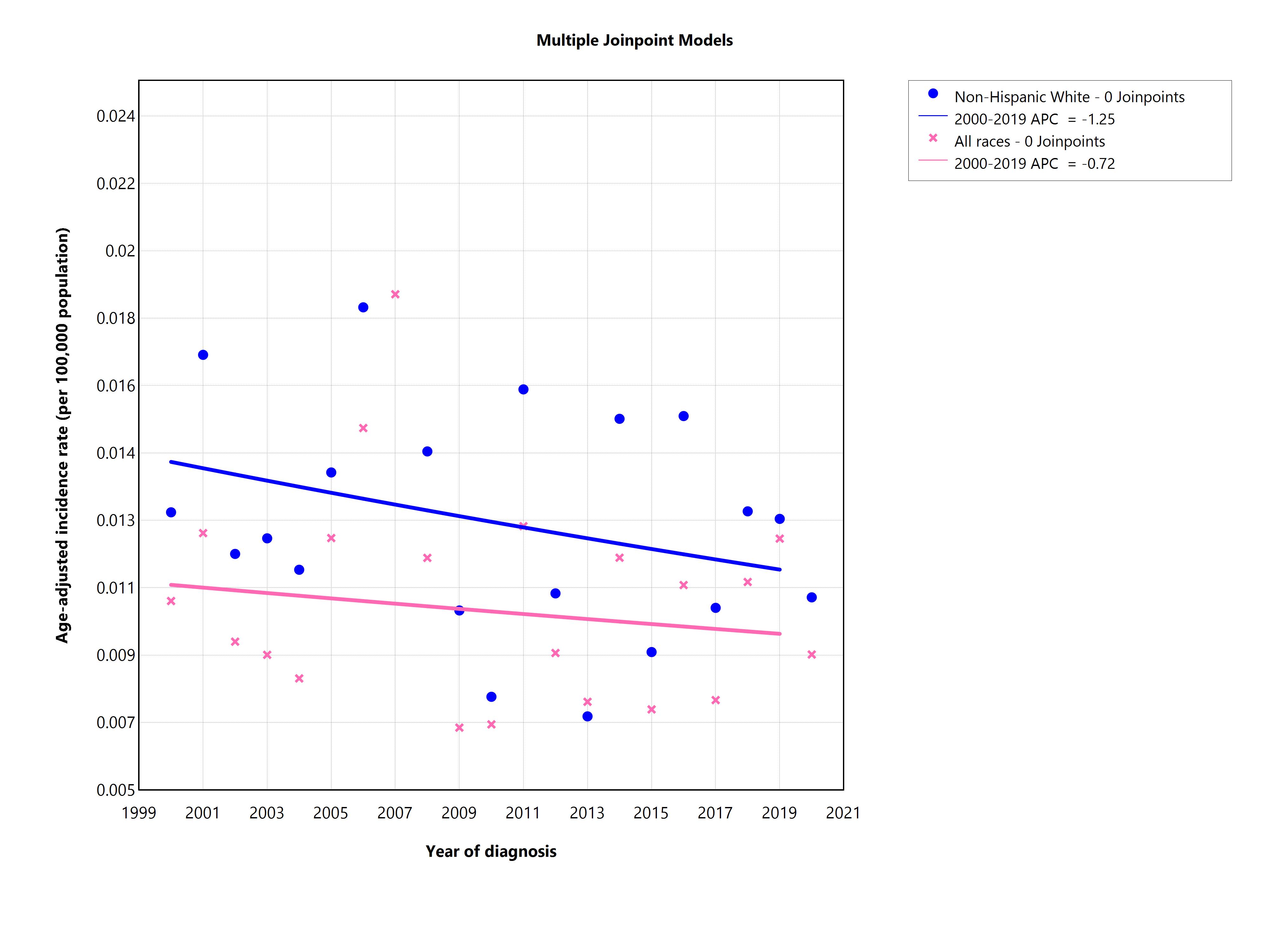


**Figure S9.** Delayed age-adjusted incidence rate of chondrosarcoma over 2000-2019 and in 2020 in the United States, by race. APC: annual percent change.


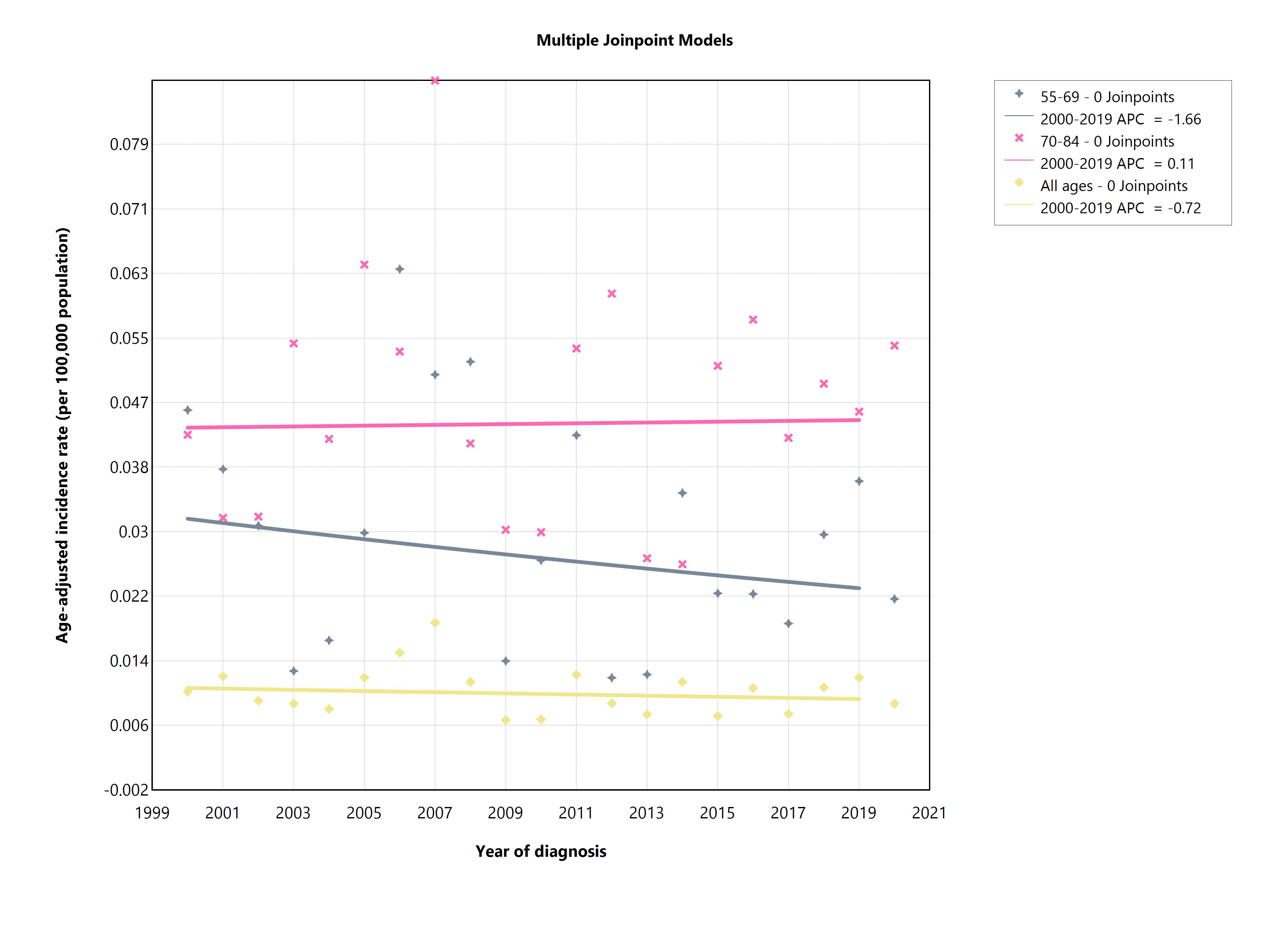


**Figure S10.** Delayed age-adjusted incidence rate of chondrosarcoma over 2000-2019 and in 2020 in the United States, by age. APC: annual percent change.


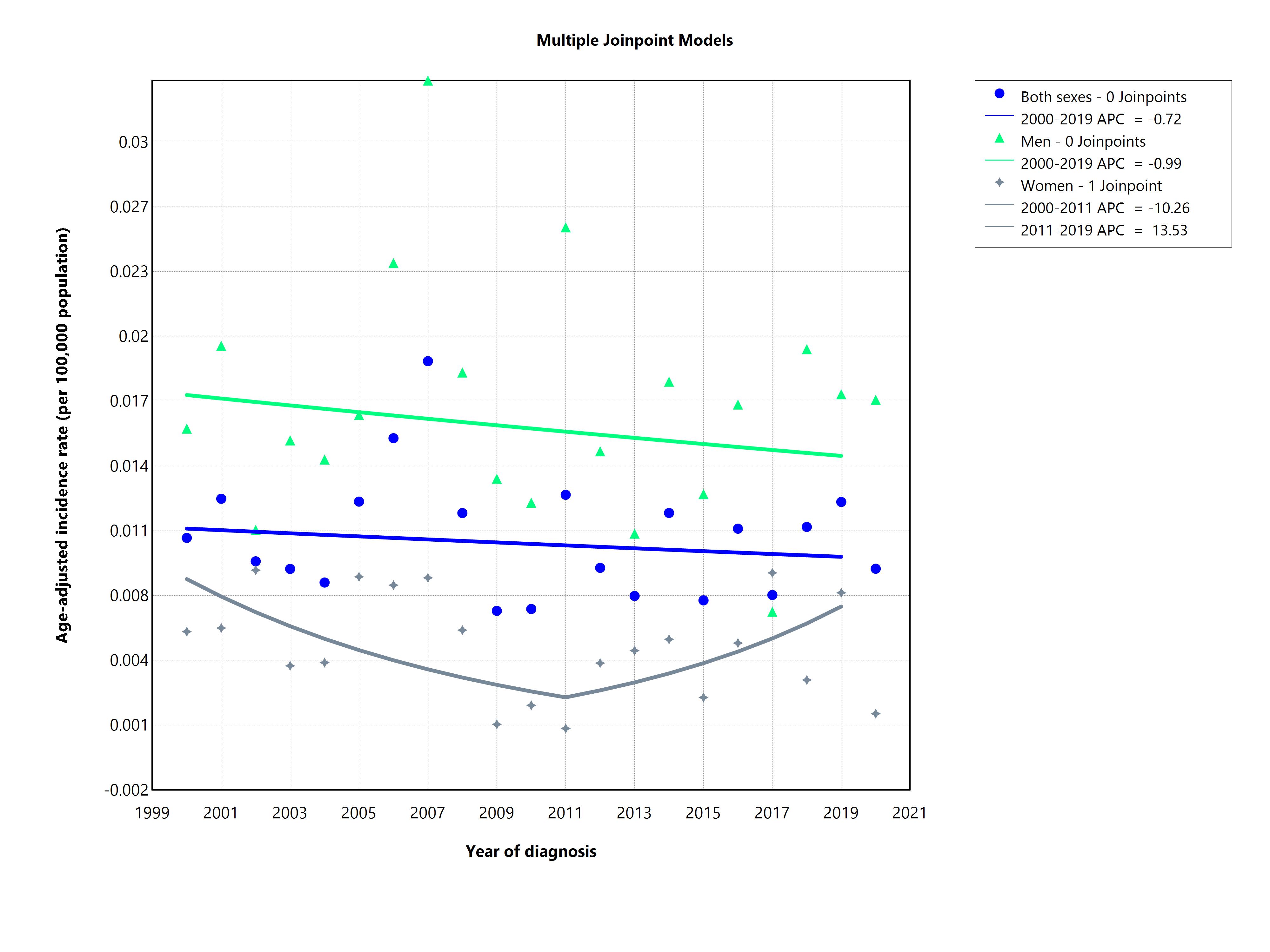


**Figure S11.** Delayed age-adjusted incidence rate of chondrosarcoma over 2000-2019 and in 2020 in the United States, by sex. APC: annual percent change.


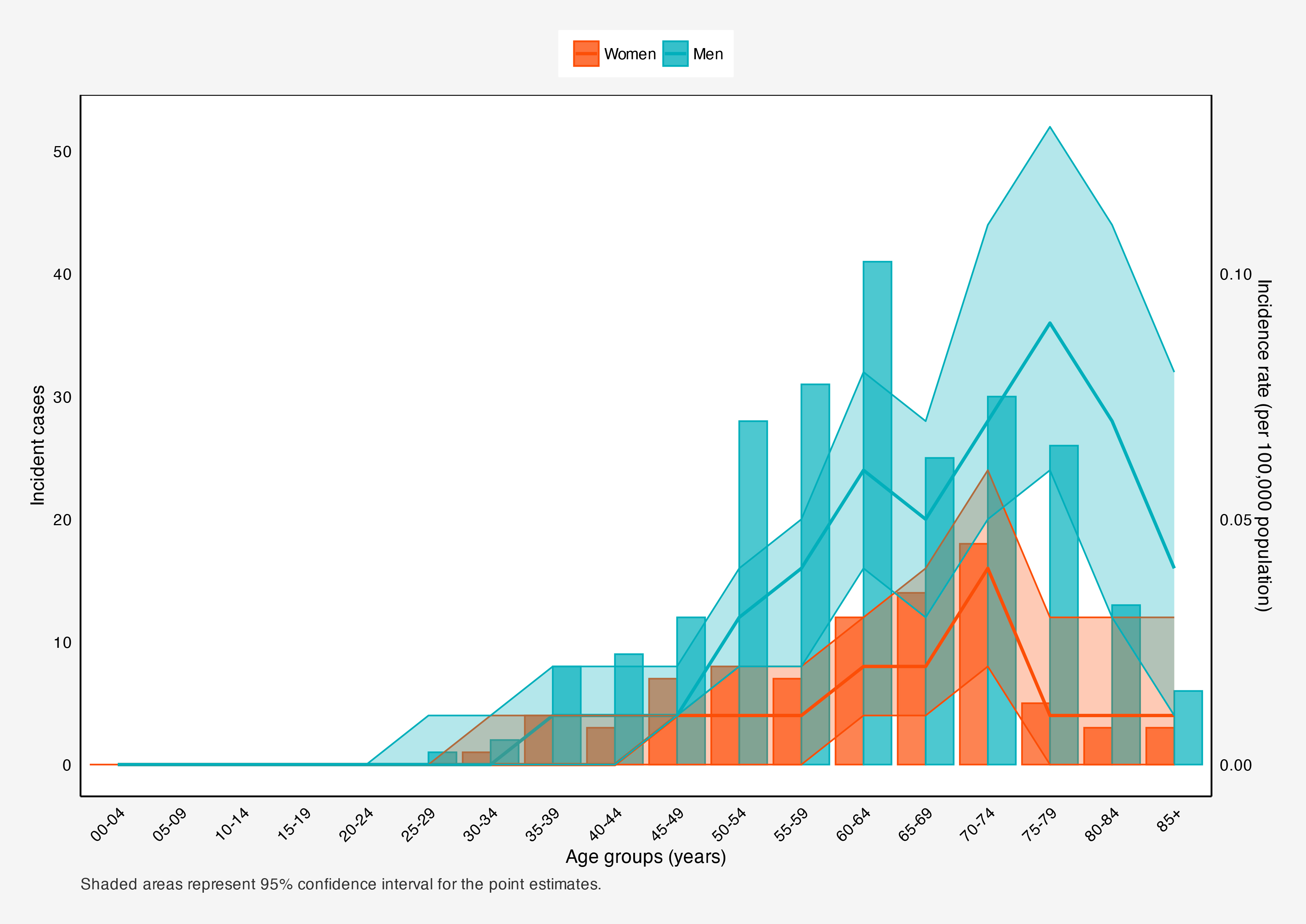


**Figure S12.** Incident cases and incidence rate of chondrosarcoma in the United States among males and females in each age group. Shaded areas are the confidence interval range for the point estimates.
